# Cost-Utility Analysis of Mycophenolate Mofetil versus Azathioprine Based Regimens for Maintenance Therapy of Proliferative Lupus Nephritis

**DOI:** 10.1155/2015/917567

**Published:** 2015-10-27

**Authors:** Robert Nee, Ian Rivera, Dustin J. Little, Christina M. Yuan, Kevin C. Abbott

**Affiliations:** Department of Nephrology, Walter Reed National Military Medical Center, 8901 Wisconsin Avenue, Bethesda, MD 20889-5600, USA

## Abstract

*Background/Aims*. We aimed to examine the cost-effectiveness of mycophenolate mofetil (MMF) and azathioprine (AZA) as maintenance therapy for patients with Class III and Class IV lupus nephritis (LN), from a United States (US) perspective. *Methods*. Using a Markov model, we conducted a cost-utility analysis from a societal perspective over a lifetime horizon. The modeled population comprised patients with proliferative LN who received maintenance therapy with MMF (2 gm/day) versus AZA (150 mg/day) for 3 years. Risk estimates of clinical events were based on a Cochrane meta-analysis while costs and utilities were retrieved from other published sources. Outcome measures included costs, quality-adjusted life-years (QALY), incremental cost-effectiveness ratios (ICER), and net monetary benefit. *Results*. The base-case model showed that, compared with AZA strategy, the ICER for MMF was $2,630,592/QALY at 3 years. Over the patients' lifetime, however, the ICER of MMF compared to AZA was $6,454/QALY. Overall, the ICER results from various sensitivity and subgroup analyses did not alter the conclusions of the model simulation. *Conclusions*. In the short term, an AZA-based regimen confers greater value than MMF for the maintenance therapy of proliferative LN. From a lifelong perspective, however, MMF is cost-effective compared to AZA.

## 1. Introduction

Lupus nephritis (LN) is a serious and costly cause of kidney disease worldwide [1]. An analysis of United States (US) medical expenditures found that the annual costs per patient among those with LN exceeded $46,000 (USD) versus matched controls and $42,000 versus systemic lupus erythematosus (SLE) patients without nephritis [2]. These findings suggest that LN is a key driver of economic burden in the SLE population.

The Kidney Disease Improving Global Outcomes (KDIGO) practice guidelines for initial or induction therapy for LN are well accepted [3]; however, they do not indicate a preference for maintenance therapy with azathioprine- (AZA-) or mycophenolate mofetil- (MMF-) based regimens. The Task Force Panel of the American College of Rheumatology recommended that either AZA or MMF be used for maintenance [4]. These recommendations were based, in large part, on two randomized controlled trials of long-term maintenance therapies for LN. In the MAINTAIN Nephritis Trial, a predominantly Caucasian cohort was randomized to MMF 2 gm/day or AZA 2 mg/kg/day as maintenance therapy after induction with a fixed, low dose intravenous (IV) cyclophosphamide (CYC) regimen [5]. After a mean follow-up of 4 years, this European-based study found that MMF was not superior to AZA in preventing renal flares, without significant differences in adverse events except for higher rate of cytopenias in the AZA group. In the larger Aspreva Lupus Management Study (ALMS) trial, a multinational population was randomized to MMF 2 gm/day or AZA 2 mg/kg/day after response to initial induction therapy [6]. After 3 years, MMF was superior to AZA as maintenance therapy, based on the primary composite end point of death, end stage renal disease (ESRD), doubling of the serum creatinine, renal flare, or requirement for rescue therapy.

To our knowledge, a cost-effectiveness analysis of maintenance therapy for proliferative LN from a US perspective has not been reported. We conducted a cost-utility analysis from a societal perspective to evaluate the cost-effectiveness of the 3-year maintenance regimens (MMF versus AZA) for proliferative LN over a lifetime horizon.

## 2. Methods

### 2.1. Study Design

We constructed a Markov state transition model to estimate the quality-adjusted life-years (QALY) and costs associated with maintenance therapy with MMF versus AZA. Markov models analyze uncertain events over time and are suited to decisions where the timing of events is important and when events are recursive in nature [7]. While decision trees model uncertain events at chance nodes, Markov models analyze these events as transitions between health states. Markov models are suited to modelling chronic conditions, where costs and outcomes (QALY) are spread over a long period of time.

Our model encompasses an initial 3-year treatment phase after which simulated patients are no longer on immunosuppressive agents and followed long term (Tables 1(a) and 2(a); Supplemental Data Sources) (see Supplementary Material available online at http://dx.doi.org/10.1155/2015/917567). The time horizon in Markov models is divided into discrete time periods, called cycles. Each cycle length in our model is 6 months for the first 3 years and 12 months thereafter, reflecting the natural history of renal flares and remissions in LN. Analyses were performed using TreeAge Pro 2012 (TreeAge Software, Williamstown, MA) and Microsoft Excel 2010 (Microsoft Corp., Redmond, WA). Institutional review board approval was not required for this study. We adhered to the CHEERS (Consolidated Health Economic Evaluation Reporting Standards) reporting guidelines in our study [8].

### 2.2. Target Population

The modeled population is a hypothetical cohort of 1,000 patients with proliferative LN receiving maintenance therapy, having responded to their induction regimen. The starting age ranges from 20 to 40 years old, and various racial/ethnic groups are represented in the model, reflecting the demographic characteristics of study participants in the clinical trials.

### 2.3. Model Structure

**Figure 1:**
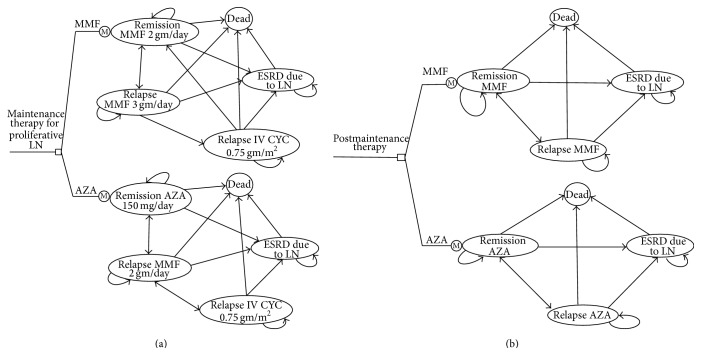
(a) Markov state transition diagram illustrating the health states and transitions for each treatment strategy for the initial 3 years. The lifetime model consists of the initial 3-year period of maintenance therapy followed by a posttreatment phase as shown in (b). (b) Markov state transition diagram illustrating the health states and transitions for each treatment strategy for the posttreatment phase (after 3 years). LN: lupus nephritis; AZA: azathioprine; MMF: mycophenolate mofetil; IV CYC: intravenous cyclophosphamide; ESRD: end stage renal disease.

The general structure of the model is shown as a state transition diagram in Figures 1(a) and 1(b) and Markov cycle trees (Supplemental Model Structure, Model Assumptions, and Supplemental Figures 1 and 2). We did not include the costs and QALY with* induction* therapy for proliferative LN given that this was a study of the differences between two maintenance treatment strategies and inclusion would not alter the conclusion of the analysis. We attempt to simulate patient-oriented outcomes and treatment strategies that are typically utilized in “real-world” clinical practice. For both strategies, after model entry each patient would progress through five potential health states, in 6-month cycles:

1. Remission state on MMF or AZA as maintenance therapy;
2. relapse of LN requiring MMF rescue therapy (escalation of MMF dose if maintained on MMF);
3. relapse of LN despite MMF rescue therapy, requiring monotherapy with IV CYC;
4. ESRD due to LN;
5. death.

Upon completing the 3-year maintenance therapy, patients in each arm are assumed to be off the immunosuppressive medications and would progress through four potential health states in the lifetime model, in 12-month cycles:

1. Remission;
2. relapse of LN;
3. ESRD due to LN;
4. death.

### 2.4. Interventions

We evaluated MMF (2 gm/day) and AZA (150 mg/day) as maintenance therapy for LN. The model accounted for sequential rescue therapy during 3 years of maintenance therapy. There is a paucity of clinical trial data on the treatment of LN flares. Therefore, the treatment approach in our model reflects the current recommendations of national and international experts [9, 10].

### 2.5. Costs

Costs of healthcare products and services were undertaken from a societal perspective. All costs were adjusted for inflation to 2013 US dollars by using the Consumer Price Index for Medical Care [11]. Drug costs are based on average wholesale prices (AWP) [12]; other cost items were obtained from previous literature and public sources. Tables 1(b) and 2(b) show the components of* direct* and* indirect* costs incurred during the 3-year maintenance therapy with either MMF or AZA and thereafter in the lifetime model (Supplemental Costs). As noted above, patients are assumed to be off immunosuppressive therapy after 3 years; therefore, costs of MMF, AZA, and CYC are not included in the lifetime model.

### 2.6. Utilities (QALY)

QALY is the product of the utility score and the number of years spent in a particular health state. A utility score reflects preference of a surveyed sample of individuals for a particular health state; a preference score of 1.0 represents perfect health, whereas a 0 score represents death. Tables 1(c) and 2(c) show the various utility weights of the health states in the model, obtained from previous literature (Supplemental Utilities).

### 2.7. Outcome Measures

The first outcome measure is the incremental cost-effectiveness ratios (ICER) which is the difference in costs between two strategies divided by the difference in effectiveness [7]:

(1) $$\begin{array}{c} ICER=\frac{\Delta C}{\Delta E}=\frac{\left(C_{1}-C_{2}\right)}{\left(E_{1}-E_{2}\right)}, \end{array}$$

where *C*_1_ is the cost of strategy 1, *C*_2_ is the cost of strategy 2, *E*_1_ is the QALY of strategy 1, and *E*_2_ is the QALY of strategy 2.

The second outcome measure is the net monetary benefit (NMB) which represents the difference between the monetary value of an incremental QALY and the cost of achieving the benefit. The strategy with the highest NMB is the most cost-effective given a WTP parameter [7]

(2) $$\begin{array}{c} NMB=\left(\;E\times \lambda \;\right)-C, \end{array}$$

where *E* is effectiveness (QALY), *λ* is WTP, and *C* is cost.

WTP is the amount society that is willing to pay for an additional QALY. We used a WTP of $50,000–$100,000 per QALY gained, often cited as the cost-effectiveness threshold in the literature [30].

### 2.8. Data Analysis

Our model is based on Reference Case analysis, a standard set of methodological practices for cost-effectiveness analysis [31]. We conducted a two-dimensional simulation via a combination of probabilistic sensitivity analysis (PSA) and microsimulation [32] (Supplemental Data Analysis). We conducted sensitivity analyses to assess uncertainty in our model (Supplemental Sensitivity Analysis). We also conducted value of information analyses, using NMB calculations from the 3-year base-case model, to estimate the expected benefit of future research [32] (Supplemental Expected Value of Perfect Information). Total costs and QALY were calculated after six 1/2-year cycles in the 3-year model and after forty 1-year cycles in the base-case lifetime model.

## 3. Results

### 3.1. Model Validation

In assessing* external validity*, we compared predicted outputs from the 3-year model with observed data, which were generally comparable and within standard deviations (Supplemental Model Validation, Assessment of External Validity, and Supplemental Table 7). We also compared simulated 10-year and 15-year survival rates from the lifetime model with actual event data [33, 34]. Overall, the predicted outcomes from the lifetime model approximated observed data from these studies (Supplemental Assessment of External Validity).

### 3.2. 3-Year Model

#### 3.2.1. Base-Case Analysis (Cochrane Data)

*(i) Cost-Effectiveness*. Compared with an AZA-based regimen, MMF had an incremental cost of $17,611 and gain of 0.0067 QALY, with an ICER of $2,630,592 per QALY (Table 3(a)).

*(ii) Sensitivity Analyses. *In a one-way sensitivity analysis, the MMF-based regimen was the favored strategy if the 6-month cost of MMF 2 gm/day was <$954.13 at WTP $50,000/QALY (Supplemental Figure  3). This is equivalent to $1.33 per 500 mg MMF tablet and represents 20.0% of the actual AWP. As shown in Table 3(b), we conducted other sensitivity analyses by excluding indirect costs, varying utility weights or changing model assumptions; the ICER (MMF versus AZA) of these analyses far exceeded the standard WTP $50,000–$100,000/QALY thresholds. 

**Figure 2:**
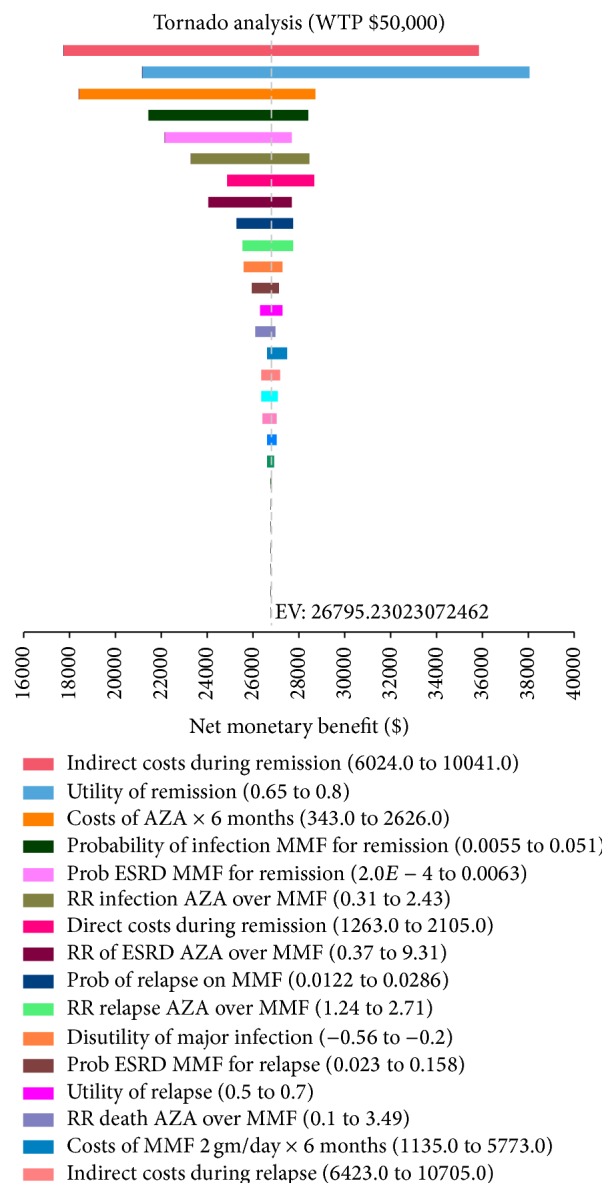
Tornado diagram of the 3-year base-case model, demonstrating one-way sensitivity analysis of each variable in the model. Each bar represents a range of expected values (EV), expressed as net monetary benefit in US dollars, over plausible estimates for an individual variable. The dotted vertical line indicates the base-case expected value. WTP: willingness-to-pay.

*(iii) Tornado Analysis*. At a WTP $50,000/QALY, the model was most sensitive to (1) indirect costs during remission; (2) utility weight of the remission state; (3) drug price of AZA 150 mg/day (Figure 2). These three parameters accounted for 82.4% of the total model uncertainty. 

*(iv) Scenario Analysis*. Despite simulated conditions biased against AZA, the MMF-based regimen remained cost ineffective compared to its alternative at 3 years, with an ICER $709,870 per QALY (Table 3(b)). 

**Figure 3:**
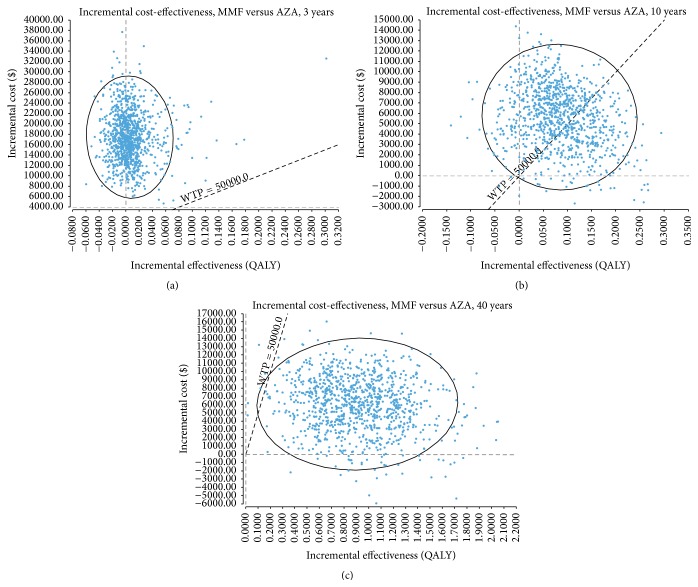
Incremental cost-effectiveness scatter plots of the base-case model. (a) 3 years; (b) 10 years after completing 3-year maintenance therapy; (c) 40 years after completing 3-year maintenance therapy. Each single point represents pairs of incremental cost and effectiveness values from probabilistic sensitivity analyses via second-order Monte Carlo simulation of 1,000 iterations. The ellipsis represents the 95% confidence interval. The dotted diagonal line represents the WTP threshold of $50,000/QALY. AZA: azathioprine; MMF: mycophenolate mofetil; WTP: willingness-to-pay; QALY: quality-adjusted life-years.

*(v) Probabilistic Sensitivity Analyses*. The incremental cost-effectiveness (ICE) scatterplot and the cost-effectiveness acceptability curve (CEAC) showed that an AZA-based regimen had a near 100% probability of being cost-effective over a 3-year time frame, at WTP thresholds of $50,000 and $100,000/QALY (Figure 3(a), Supplemental Figure 4). 

*(vi) Expected Value of Perfect Information (EVPI)*. The* population* EVPI represents the upper bound on the expected gain on investment on further data collection, which we calculated to be $2,058,206 at WTP $100,000/QALY in the US population, assuming a period of 10 years with 3% discount rate (Supplemental Expected Value of Perfect Information). 

#### 3.2.2. Subgroup Analysis

Based on ALMS data, MMF had an ICER of $700,001 per QALY compared with an AZA-based regimen (Table 3(a)). Furthermore, AZA was both cost-saving and more effective than MMF using data from the MAINTAIN trial (Table 3(a)).

### 3.3. Lifetime Model (40 Years)

#### 3.3.1. Base-Case Analysis (Cochrane Data)

*(i) Cost-Effectiveness*. Compared with an AZA-based regimen, MMF had an incremental cost of $5,976 and gain of 0.9260 QALY, with an ICER of $6,454 per QALY (Table 4(a)).

*(ii) Probabilistic Sensitivity Analyses*. The CEAC showed that an MMF-based regimen had a near 100% probability of being cost-effective over a 40-year time frame, at WTP thresholds of $50,000 and $100,000/QALY (Supplemental Figure  5). 

*(iii) Sensitivity Analyses*. As shown in Table 4(b), the ICER (MMF versus AZA) decreased over time such that MMF became cost-effective compared to AZA at 10 years postmaintenance therapy (Figures 3(b) and 3(c)). We also conducted sensitivity analyses by varying the probability of ESRD in the relapse state, demonstrating that the higher the risk of ESRD, the greater the cost-effectiveness of MMF versus AZA. Given the higher baseline risk of ESRD on AZA maintenance therapy, any incremental increase in this risk would disproportionately affect AZA (higher costs and lower QALY) as compared to MMF, resulting in a lower ICER (MMF versus AZA). We conducted other sensitivity analyses by excluding indirect costs, varying utility weights or discount rates, with the ICER (MMF versus AZA) well below the WTP $50,000/QALY threshold (Table 4(b)). 

*(iv) Scenario Analysis*. The ICER of the base-case ($6,454/QALY) was based on the assumption that the treatment effect of MMF and AZA during the trial phase would persist over a lifetime. As shown in Table 4(b), MMF remained cost-effective over lifetime even if the treatment effect of both therapies diminished by 1% or 2% per year. However, assuming no treatment benefit after 3 years of maintenance therapy with either agent, MMF was not cost-effective compared to AZA ($428,894/QALY).

#### 3.3.2. Subgroup Analysis

MMF had favorable ICER compared to AZA over lifetime using ALMS ($4,394/QALY) and MAINTAIN data ($54,891/QALY), below the WTP $50,000–$100,000/QALY (Table 4(a)).

## 4. Discussion

MMF and AZA are the most widely used therapeutic agents for long-term maintenance therapy of proliferative LN [35]. However, there is no consensus on the agent of choice, reflected by current clinical practice guideline recommendations [3, 4]. To evaluate the cost-effectiveness of MMF versus AZA-based regimens, we developed a Markov model to simulate patient-oriented outcomes, both from short-term and from lifetime horizon.

We found poor cost-effectiveness of MMF versus AZA-based therapy at 3 years, with an ICER $2,630,592/QALY. The ICER of MMF versus AZA remained substantially elevated in sensitivity analyses, even in conditions biased against AZA. Over a lifetime, however, our base-case analysis demonstrated MMF to be cost-effective compared to AZA, with an ICER $6,454/QALY. Overall, the ICER results from various sensitivity analyses did not alter the conclusions of the lifetime model, except in an unlikely scenario where the treatment effect was nil after 3 years of maintenance therapy. In contrast to the initial 3-year time period, subgroup analysis of ALMS and MAINTAIN trials showed that the MMF-based strategy was cost-effective compared to AZA from a lifetime perspective, at WTP $50,000–$100,000/QALY.

To our knowledge, there are only two published cost-effectiveness analyses of LN treatment. Wilson et al. estimated the cost-utility of MMF versus IV CYC as induction therapy for 6 months from the perspective of the National Health Service in the United Kingdom (UK) [36]. Their analysis suggested that MMF was likely to result in better quality of life and be less expensive than IV CYC as induction therapy. More recently, Mohara et al. conducted a lifetime cost-utility analysis of four different immunosuppressive regimens for LN patients in Thailand [37]. The study demonstrated that, from a Thai perspective, induction with IV CYC followed by AZA was the most cost-effective regimen of all the alternatives. Our study reached different conclusions due to notable mutual differences in the model structure and assumptions, setting (US versus Thailand), target population, cost and utility parameters, and transition probabilities; and the Cochrane meta-analysis was not used as data source in Mohara's study.

Our study has certain limitations. First, lifetime modeling required extrapolation of data beyond the period observed in clinical trials which could lead to inconsistent results. We therefore assessed the uncertainty of future treatment benefit by conducting sensitivity analyses based on established guidelines [38]. Second, we assume that patients in our model receive immunosuppressive maintenance therapy for 3 years based on published clinical trials [13]. Due to lack of data from long-term randomized studies of maintenance therapy in patients with proliferative lupus nephritis, we did not model scenarios whereby patients are kept on maintenance therapy for >3 years. Modeling such scenarios based on incomplete medical evidence would compromise face validity. Furthermore, we would not be able to test the model for external validity (comparing predicted results from the model with actual event data) [39]. Third, the* total* costs of each strategy are likely underestimated since cost data are based on the Tri-Nation Study which included lupus patients from the US, Canada, and UK [18, 19]. This study estimated that direct and indirect costs in the US are 20% and 29% higher, respectively, than Canada. However, this underestimation of total costs does not change the conclusions of our analysis which is based on incremental calculations. Fourth, our model included utility scores that were measured by VAS [17, 23] which does not involve a trade-off that a subject must choose between the health states, in contrast to the standard gamble and time trade-off techniques. However, VAS was demonstrated to be a valid and reliable measure of health related quality of life in a SLE cohort [40]. Lastly, we incorporated major infection in the model as the most severe side effect of immunosuppressive therapy but did not consider gastrointestinal disturbance, leukopenia, alopecia, or infertility.

Acknowledging these limitations, our study does suggest that, from a cost-effectiveness standpoint, an AZA-based regimen confers greater value than MMF for the maintenance therapy of proliferative LN in the short term. Value of information analysis suggests a population EVPI of $2,058,206 at WTP $100,000/QALY which represents the expected maximum gain on investment on further research. The implication is that spending more than this amount on additional data collection would represent a poor investment of limited research funds. In contrast to the short-term perspective, MMF is cost-effective compared to AZA at the standard WTP threshold in the US over the patients' lifetime. Despite the relatively higher upfront costs of MMF during the 3-year maintenance phase, its salutary effects (lower risk of LN relapse and progression to ESRD compared to AZA) make MMF a cost-effective option over the long term. Our analysis is consistent with the general notion that the time frame of a model should be sufficiently long to capture future differences in costs and health outcomes between treatment strategies.

Given the substantial economic burden of LN in our healthcare system, the findings of this study should be an important factor in selecting the optimal maintenance regimen for patients with proliferative LN. Furthermore, these findings may provide useful information to support more individualized therapy.

## Supplementary Material

The following Supplementary Material includes additional text, tables and figures that are not presented in the main manuscript. The document provides more detailed and technical descriptions of the models and data analysis.

## Figures and Tables

**(a):** 

Probability parameters (over 6-month period or one cycle)^a^	Mean	Range (95% CI)	Probability distribution^b^	Sources
Remission AZA				
Probability of lupus-related death during remission	0.0025	0.0004–0.0157	Beta (24.3, 9830.3)	Cochrane 2012 [13]
Probability of major infection during remission	0.0138	0.0047–0.0430	Beta (751.2, 53,686.0)	Cochrane 2012 [13]
Probability of ESRD during remission	0.0030	0.0006–0.0160	Beta (35.9, 11,927.1)	Cochrane 2012 [13]
Probability of relapse during remission	0.0364	0.0234–0.0587	Beta (5106, 135,192.3)	Cochrane 2012 [13]
Remission MMF				
Probability of lupus-related death during remission	0.0043	0.0007–0.0285	Beta (4.2, 963.5)	Cochrane 2012 [13]
Probability of major infection during remission	0.0160	0.0055–0.0510	Beta (3.9, 241.1)	Cochrane 2012 [13]
Probability of ESRD during remission	0.0012	0.0002–0.0063	Beta (3.9, 3269.0)	Cochrane 2012 [13]
Probability of relapse during remission	0.0185	0.0122–0.0286	Beta (4.1, 219.5)	Cochrane 2012 [13]
Relapse MMF (2 gm/d or 3 gm/d)				
Probability of lupus-related death during relapse	0.0410	0.0210–0.0790	Beta (64.4, 1507.3)	Cochrane 2012 [13]
Probability of major infection during relapse	0.1210	0.0810–0.1830	Beta (514.6, 3738.7)	Cochrane 2012 [13]
Probability of ESRD during relapse	0.0610	0.0230–0.1580	Beta (139.7, 2150.5)	Cochrane 2012 [13]
Probability of complete and partial remissions	0.5900	0.4180–0.7380	Beta (56.5, 39.3)	Cochrane 2012 [13]
Relapse CYC				
Probability of lupus-related death during relapse	0.0400	0.0200–0.0780	Beta (61.4, 1473.6)	Cochrane 2012 [13]
Probability of major infection during relapse	0.1090	0.0730–0.1650	Beta (105.8, 864.4)	Cochrane 2012 [13]
Probability of ESRD during relapse	0.0855	0.0320–0.2220	Beta (66.8, 714.1)	Cochrane 2012 [13]
Probability of complete and partial remissions	0.5220	0.3920–0.6520	Beta (51.6, 47.2)	Cochrane 2012 [13]
ESRD due to lupus nephritis				
Probability of death due to lupus nephritis ESRD	0.0513	0.0481–0.0548	Beta (99.8, 1845.9)	Costenbader et al. 2011 [14]

AZA: azathioprine; MMF: mycophenolate mofetil; CYC: cyclophosphamide; ESRD: end stage renal disease; CI: confidence interval.

^a^Probabilities from the data sources were reported over various follow-up durations. *Probabilities* were converted to *rates* and then to 6-month probabilities [15]. First, the probabilities were converted to yearly rates (event per patient per year) using the equation *r* = − (1/*t*)ln⁡(1 − *P*), where *r* = rate; *t* = time in years; *P* = probability of an event occurring during time *t*.

These annual rates were then converted to 6-month probabilities using the equation *P* = 1 − *e* ^−*rt*^, where *r* = one-year rate; *t* = time in years; *P* = probability of an event occurring during time *t*.

^b^Beta distributions are characterized by (*α*, *β*).

**(b):** 

Cost parameters (over 6-month period)	Mean costs ($)	Range ($)	Probability distribution^g^	Sources/Comments
AZA 150 mg/day × 6 months^a^	769.86	343.98–2626.26	Gamma (59.1, 0.08)	Red Book 2013 [12]
MMF 2000 mg/day × 6 months^b^	4833.92	1135.68–5773.04	Gamma (23.4, 0.005)	Red Book 2013 [12]
MMF 3000 mg/day × 6 months^b^	7250.88	1703.52–8659.56	Gamma (52.6, 0.007)	Red Book 2013 [12]
Monthly infusion of CYC 0.75 gm/m^2^ × 6 months to treat LN relapse^d^	6233.52^c^	4675.14–7791.90	Gamma (42.1, 0.006)	Red Book 2013 [12], CMS 2013 [16]; range assumed to be ±25% mean
Direct costs × 6 months (nonpharmaceuticals)^e^				
Remission	1684.17	1263.13–2105.21	Gamma (31.5, 0.019)	Clarke et al. 2008 [17]; Clarke et al. 2004 [18]; range assumed to be ±25% mean
Relapse	3243.43	2432.57–4054.29	Gamma (29.2, 0.009)	Clarke et al. 2008 [17]; Clarke et al. 2004 [18]; range assumed to be ±25% mean
Indirect costs × 6 months^f^				
Remission	8033.19	6024.89–10041.49	Gamma (16.1, 0.002)	Clarke et al. 2008 [17]; Panopalis et al. 2007 [19]; range assumed to be ±25% mean
Relapse	8564.07	6423.05–10705.09	Gamma (18.3, 0.002)	Clarke et al. 2008 [17]; Panopalis et al. 2007 [19]; range assumed to be ±25% mean
ESRD/dialysis: mean cost per person × 6 months	43,304	n/a	Gamma (75.0, 0.002)	USRDS 2012 [20]
Major infection (inpatient cost for septicemia, ICD9 code 038.9)	17,183	16,849–17,517	Gamma (32.8, 0.002)	Healthcare Cost and Utilization Project [21]

AZA: azathioprine; MMF: mycophenolate mofetil; CYC: cyclophosphamide (intravenous); ESRD: end stage renal disease; USRDS: United States Renal Data System; LN: lupus nephritis.

^a^Based on unit cost of AZA 50 mg tablet = $1.41 (range 0.63–4.81) [12].

^b^Based on unit cost of MMF 500 mg tablet = $6.64 (range 1.56–7.93) [12].

^c^Based on monthly cost of intravenous CYC 0.75 gm/m^2^  = $1038.92 [12, 16].

^d^See Supplemental Table 6: costs of individual components of intravenous cyclophosphamide infusion.

^e^Components of direct costs included care provided by specialists, nonspecialists, nonphysician healthcare professionals, laboratory studies, imaging studies, emergency room visits, outpatient surgery, and hospitalizations [18].

^f^Indirect costs included time lost from labor and nonlabor (i.e., household work) market activity as well as time that a caregiver spent helping the patient receiving healthcare services and the time the caregiver spent doing housework [19].

^g^Gamma distributions are characterized by (*α*, *λ*); *α* = *μ* ^2^/*s* ^2^, *λ* = μ/*s* ^2^, where *μ* = mean; *s* ^2^ = variance.

**(c):** 

Utility parameters	Base-case mean	Range^b^	Probability distribution^c^	Sources/Comments
Utility of ESRD on dialysis	0.67	0.54–0.85	Beta (14.1, 6.9)	Liem et al. 2008 [22], based on TTO method
Utility of remission, on MMF or AZA	0.70	0.65–0.80	Beta (14.0, 6.0)	Grootscholten et al. 2007 [23], Clarke et al. 2008 [17], based on VAS method
Utility of relapse, requiring MMF rescue therapy	0.60	0.50–0.70	Beta (13.8, 9.2)	Grootscholten et al. 2007 [23], Clarke et al. 2008 [17], based on VAS method
Utility of relapse, requiring CYC rescue therapy	0.50	0.40–0.60	Beta (12.0, 12, 0)	Tse et al. 2006 [24]; requiring CYC after failing MMF rescue therapy
Disutility^a^ of major infection (sepsis)	0.31	0.20–0.56	Fixed	Cost-Effectiveness Analysis Registry 2013 [25]
Utility of death	0.00	n/a	n/a	Drummond et al. 2005 [26]

AZA: azathioprine; MMF: mycophenolate mofetil; CYC: cyclophosphamide (intravenous); ESRD: end stage renal disease; TTO: time trade-off; VAS: visual analog scale.

^a^Disutility = 1 − utility weight.

^b^Based on 95% confidence interval or standard deviation.

^c^Beta distributions are characterized by (*α*, *β*).

**(a):** 

Probability parameters (1-year cycle)^a^	Base-case value	Range (95% CI)	Probability distribution^b^	Sources
Remission in AZA group				
Probability of lupus-related death during remission	Age-dependent^g^	n/a	n/a	Bernatsky et al. 2006 [27]; Arias 2011 [28]; Cochrane 2012 [13]
Probability of ESRD during remission	0.0061	0.0012–0.0317	Beta (35.9, 11,927.1)	Cochrane 2012 [13]
Probability of relapse during remission	0.0716	0.0463–0.1140	Beta (5106.9, 135,192.3)	Cochrane 2012 [13]
Remission in MMF group				
Probability of lupus-related death during remission	Age-dependent^f^	n/a	n/a	Bernatsky et al. 2006 [27]; Arias 2011 [28]; Cochrane 2012 [13]
Probability of ESRD during remission	0.0025	0.0005–0.0125	Beta (16.6, 6771.3)	Cochrane 2012 [13]
Probability of relapse during remission	0.0367	0.0244–0.0564	Beta (16.0, 419.5)	Cochrane 2012 [13]
Relapse in MMF group				
Probability of lupus-related death during relapse	Age-dependent^e^	n/a	n/a	Bernatsky et al. 2006 [27]; Arias 2011 [28]
Probability of ESRD during relapse	0.1183	0.0455–0.2910	Beta (491.1, 3670.9)	Cochrane 2012 [13]
Probability of complete and partial remissions	0.8319	0.6613–0.9313	Beta (45.7, 9.2)	Cochrane 2012 [13]
Relapse in AZA group				
Probability of lupus-related death during relapse	Age-dependent^e^	n/a	n/a	Bernatsky et al. 2006 [27]; Arias 2011 [28]
Probability of ESRD during relapse	0.1183^c^	0.0455–0.2910	Beta (491.1, 3670.9)	Cochrane 2012 [13]
Probability of complete and partial remissions	0.8319^c^	0.6613–0.9313	Beta (45.7, 9.2)	Cochrane 2012 [13]
ESRD due to lupus nephritis				
Probability of death due to lupus nephritis ESRD	Age-dependent^d^	n/a	n/a	USRDS 2012 [20]; Sule et al. 2011 [29]

AZA: azathioprine; MMF: mycophenolate mofetil; CYC: cyclophosphamide; ESRD: end stage renal disease; CI: confidence interval.

^a^Probabilities from the data sources were reported over various follow-up durations. *Probabilities* were converted to *rates* and then to 6-month probabilities [15]. First, the probabilities were converted to yearly rates (event per patient per year) using the equation *r* = − (1/*t*)ln⁡(1 − *P*), where *r* = rate; *t* = time in years; *P* = probability of an event occurring during time *t*.

These annual rates were then converted to 6-month probabilities using the equation *P* = 1 − *e* ^−*rt*^, where *r* = one-year rate; *t* = time in years; *P* = probability of an event occurring during time *t*.

^b^Beta distributions are characterized by (*α*, *β*).

^c^Probability based on MMF for relapse in either AZA- or MMF-based regimen.

^d^The age-specific annual mortality rate for the general dialysis population in 2011 [20] is multiplied by hazard ratio (HR) 1.7. In a USRDS study, Sule et al. found that adult patients with ESRD secondary to SLE were at increased risk of death compared with other adult patients (HR 1.7; 95% CI 1.2–2.7) [29]. Conversion between rates and probabilities as noted above.

^e^In the relapse state for both MMF and AZA strategies, the rate of lupus-related death is derived from age-specific annual mortality rate in the general population [28] multiplied by a standardized mortality ratio (SMR) 7.9. In a cohort of 9,547 SLE patients, Bernatsky et al. estimated an SMR 7.9 in those with nephritis [27]. Conversion between rates and probabilities as noted above.

^f^Values in (e) divided by 9.3, given that the relative risk of lupus-related death during relapse versus remission on MMF treatment is 9.3 [13].

^g^Values in (f) × 0.58, given that the relative risk of lupus-related death during remission on AZA versus MMF is 0.58 [13].

**(b):** 

Cost parameters (over 1-year period)	Mean costs ($)	Range ($)	Probability distribution^c^	Sources/Comments
Direct costs × 1 year (nonpharmaceuticals)^a^				
Remission	3,368.34	1263.13–2105.21	Gamma (31.5, 0.019)	Clarke et al. 2004 [18]; Clarke et al. 2008 [17]; range assumed to be ±25% mean
Relapse	6,486.85	2432.57–4054.29	Gamma (29.2, 0.009)	Clarke et al. 2004 [18]; Clarke et al. 2008 [17]; range assumed to be ±25% mean
Indirect costs × 1 year^b^				
Remission	16,066.38	6024.89–10041.49	Gamma (16.1, 0.002)	Panopalis et al. 2007 [19]; Clarke et al. 2008 [17]; range assumed to be ±25% mean
Relapse	17,128.13	6423.05–10705.09	Gamma (18.3, 0.002)	Panopalis et al. 2007 [19]; Clarke et al. 2008 [17]; range assumed to be ±25% mean
ESRD/dialysis: mean cost per person × 1 year	86,608	n/a	Gamma (75.0, 0.002)	USRDS 2012 [20]

ESRD: end stage renal disease; USRDS: United States Renal Data System.

^a^Direct costs included care provided by specialists, nonspecialists, nonphysician healthcare professionals, laboratory studies, imaging studies, emergency room visits, and outpatient surgery and hospitalizations [18].

^b^Indirect costs included time lost from labor and nonlabor (i.e., household work) market activity as well as time that a caregiver spent helping the patient receiving healthcare services and the time the caregiver spent doing housework [19].

^c^Gamma distributions are characterized by (*α*, *λ*); *α* = *μ* ^2^/*s* ^2^, *λ* = μ/*s* ^2^, where *μ* = mean; *s* ^2^ = variance.

**(c):** 

Utility parameters	Base-case mean	Range^a^	Probability distribution^b^	Sources/Comments
Utility of ESRD on dialysis	0.67	0.54–0.85	Beta (14.1, 6.9)	Liem et al. 2008 [22], based on TTO method
Utility of remission, on MMF or AZA	0.70	0.65–0.80	Beta (14.0, 6.0)	Grootscholten et al. 2007 [23], Clarke et al. 2008 [17], based on VAS method
Utility of relapse, on MMF or AZA	0.60	0.50–0.70	Beta (13.8, 9.2)	Grootscholten et al. 2007 [23], Clarke et al. 2008 [17], based on VAS method
Utility of death	0.00	n/a	n/a	Drummond et al. 2005 [26]

AZA: azathioprine; MMF: mycophenolate mofetil; ESRD: end stage renal disease; TTO: time trade-off; VAS: visual analog scale.

^a^Based on 95% confidence interval or standard deviation.

^b^Beta distributions are characterized by (*α*, *β*).

**(a):** 

Scenarios	Total cost ($)	Total effectiveness (QALY)	Incremental costs ($)	Incremental effectiveness (QALY)	ICER ($/QALY)
Cochrane (base-case)					
AZA	54,249.98	1.6367			
MMF	71,861.21	1.6434	17,611.23	0.0067	2,630,591.76
Subgroups					
ALMS					
AZA	55,959.12	1.6125			
MMF	72,619.05	1.6363	16,659.92	0.0238	700,001.12
MAINTAIN					
AZA	54,527.62	1.6318			
MMF	72,511.65	1.6148	17,984.04	−0.0170	Dominated

QALY: quality-adjusted life-years; AZA: azathioprine; MMF: mycophenolate mofetil.

**(b):** 

Scenarios	ICER MMF versus AZA (US$)
Base-case	2,630,591.76
Excludes indirect costs in both strategies	2,529,609.93
*Utility*	
Remission = 0.8 (versus base-case 0.7)	1,476,631.93
Relapse requiring MMF = 0.5 (versus base-case 0.6)	1,654,369.09
Utility of relapse requiring CYC = utility of relapse requiring MMF rescue	2,555,137.00
*Conditions biased against AZA-based strategy*	
Indirect costs × 6 months during remission ($10,041.49) [higher indirect costs for AZA group]	2,410,632.95
Indirect costs × 6 months during remission ($10,041.49) + utility of remission state (0.8) [higher indirect costs for AZA group + higher utility during remission]	1,380,997.67
Indirect costs × 6 months during remission ($10,041.49) + utility of remission state (0.8) + drug costs of AZA × 6 months ($2626) [higher indirect costs for AZA group + higher utility during remission + higher drug costs of AZA]	709,870.18
*Revised assumptions*	
AZA group receives 3 gm/day of MMF as rescue (base-case 2 gm/day MMF)	1,900,694.28
Patients in the AZA group who remit on CYC rescue therapy are treated with AZA maintenance therapy (base-case MMF 2 gm/day)	2,273,422.51

ICER: incremental cost effectiveness ratio; AZA: azathioprine; MMF: mycophenolate mofetil.

**(a):** 

Scenarios	Total cost ($)	Total effectiveness (QALY)	Incremental costs ($)	Incremental effectiveness (QALY)	ICER ($/QALY)
Cochrane (base-case)					
AZA	478,333.42	14.1623			
MMF	484,309.78	15.0882	5976.36	0.9260	6454.24
Subgroups					
ALMS					
AZA	485,791.18	13.5979			
MMF	493,953.07	15.4554	8161.89	1.8575	4393.90
MAINTAIN					
AZA	469,825.11	14.0140			
MMF	486,758.11	14.3225	16,933.00	0.3085	54,891.42

QALY: quality-adjusted life-years; AZA: azathioprine; MMF: mycophenolate mofetil.

**(b):** 

Scenarios	ICER MMF versus AZA (US$)
Base-case (40-year time horizon)	$6,454.24
Excluding indirect costs	Dominant^a^
Utility	
Remission 0.8 (versus base-case 0.7)	$4067.55
Relapse 0.5 (versus base-case 0.6)	$5,808.27
Relapse 0.7 (versus base-case 0.6)	$7,695.58
Increase in probability of ESRD with relapse	
0.5% per year	$4590.37
1.0% per year	$3112.96
2.0% per year	$2717.08
Extrapolated treatment effect after 3-year maintenance therapy	
Same as during treatment phase (base-case)	$6,454.24
No treatment effect from both MMF and AZA during extrapolated phase^b^	$428,894.16
Treatment effect from both MMF and AZA decreases 1% per year^c^	$15,096.38
Treatment effect from both MMF and AZA decreases 2% per year^c^	$25,713.36
Time horizon (number of years after maintenance therapy)	
5 years	$513,712.88
10 years	$67,203.94
20 years	Dominant^a^
30 years	$5,232.11
Discount rate (base-case 3% for costs and utility)	
0%	$5,830.11
5%	$10,230.91
7%	$14,374.62

ICER: incremental cost effectiveness ratio; AZA: azathioprine; MMF: mycophenolate mofetil; ESRD: end stage renal disease.

^a^MMF is less costly and more effective than AZA-based regimen.

^b^Assuming 100% probability of relapse during remission on either MMF or AZA after completing 3-year maintenance therapy.

^c^Assuming 1% or 2% per year increase in relapse during remission on either MMF or AZA after completing 3-year maintenance therapy.
