# Supplementary material for: Cost-Utility Analysis of Mycophenolate Mofetil versus Azathioprine Based Regimens for Maintenance Therapy of Proliferative Lupus Nephritis
Source: Int J Nephrol. 2015 Oct 27;2015:917567. doi: 10.1155/2015/917567 (PMC4639665; doi:10.1155/2015/917567)
Supplement: Supplementary file 1 — The following Supplementary Material includes additional text, tables and figures that are not presented in the main manuscript. The document provides more detailed and technical descriptions of the models and data analysis. [file 917567.f1.docx]

Model Structure

It is assumed that all simulated patients start in the remission state for each strategy, for the first 6 months. Upon entering each non-ESRD health state, patients could die as a result of lupus-related causes, due to complications from the underlying disease or from immunosuppressive therapy. Probabilities of lupus-related death varied among different health states in both treatment arms. Patients could also die from causes unrelated to lupus, the latter based on published life tables of age-specific mortality rate in the US general population^[[1]](#endnote-1)^.

Survivors could develop a major infection, defined as serious all-cause infections (e.g. sepsis) excluding herpes zoster, resulting in disutility and additional costs, or progress to ESRD. Patients not developing ESRD either stayed in remission or could relapse while on maintenance therapy. Patients who relapse on AZA (2mg/kg/day) maintenance therapy would be treated with MMF (2mg/day) rescue therapy and then if necessary monthly IV CYC (0.75gm/m^2^) over the 6-month cycle. Patients relapsing on MMF maintenance therapy (2gm/day) would be treated with a higher dose of MMF (3gm/day) and then with IV CYC if necessary. Therefore the two strategies are not mutually exclusive.

Patients entering the post-maintenance phase are assumed to be in the remission state. Patients in remission could die from lupus or other causes, while survivors who do not develop ESRD would either stay in remission or relapse. Those who enter and subsequently survive the relapse state can either remit or stay in relapse. The number of patients in the ESRD or dead state would accrue over time, the latter being the absorbing state. In our base-case lifetime model, the Markov simulation terminates at 40 years subsequent to the initial 3-year maintenance period.

Model Assumptions

1. Maintenance therapy (1^st^ three years)
   1. Every simulated patient has achieved remission with induction therapy prior to entering the model and therefore starts in the remission health state in both treatment arms.
   2. Every simulated patient who relapses undergoes rescue therapy.
   3. For each strategy, simulated patients who are treated with MMF for a total of 3 relapses (over 3 cycles or 18 months) are transitioned to IV CYC. Patients can only receive up to two 6-month cycles of IV CYC for relapse prior to cessation of the 3-year model, with accrued costs and quality-adjusted life-years (QALYs) associated with the relapse IV CYC state:
      1. Cycle 1 (6 months): in remission
      2. Cycle 2 (12 months): first relapse (treated with MMF 3gm/d in MMF group or MMF 2gm/d in AZA group)
      3. Cycle 3 (18 months): second relapse (treated with MMF 3gm/d in MMF group or MMF 2gm/d in AZA group)
      4. Cycle 4 (24 months): third relapse (treated with MMF 3gm/d in MMF group or MMF 2gm/d in AZA group)
      5. Cycle 5 (30 months): fourth relapse (treated with IV CYC for both groups)
      6. Cycle 6 (36 months): fifth relapse (treated with IV CYC for both groups)
   4. In all relapse states in the model, the probabilities of events (lupus-related death, major infection, end-stage renal disease [ESRD] and subsequent relapse) are based on clinical studies of induction therapy in “fresh” patients with proliferative lupus nephritis (LN)^[[2]](#endnote-2)^.
   5. Doses of medications do not change within each cycle.
2. Post maintenance therapy (after three years)
   1. Simulated patients are off immunosuppressive therapy with MMF or AZA after 3 years.
   2. Simulated patients entering the post-maintenance phase are assumed to be in remission for both treatment arms.
   3. The treatment effect of MMF and AZA during the trial phase would persist over lifetime in the base-case model (see Table 4(b) in the main manuscript for sensitivity analyses on the extrapolated treatment effect).
   4. The probabilities of achieving remission or developing ESRD during the relapse state for both strategies are based on treatment of relapse with MMF.

Data Sources

Risk estimates of clinical events (risks of death from lupus, major infection, ESRD and relapse of LN) were based on a recent systematic review and random-effects meta-analysis conducted by the Cochrane Renal Group^2^, as shown in Tables 1(a) & 2(a) (Supplemental- Tables 1, 2, 5, 8, 9). This Cochrane meta-analysis of maintenance therapy with MMF vs. AZA was based on 3 clinical trials (MAINTAIN, ALMS and Contreras’s study) and was the foundation for our base-case model. Conducting our own formal data synthesis was therefore unnecessary***.*** Given the heterogeneity in the clinical and demographic characteristics of the patient population among these clinical trials, we also conducted simulations using risk estimates from the ALMS and MAINTAIN trials to evaluate subgroup-specific cost effectiveness (Supplemental-Tables 3, 4, 10, 11). We did not conduct subgroup analysis using data from Contreras’s study^[[3]](#endnote-3)^ due to its small sample size.

Costs

Clarke et al reported that increase in renal damage was associated with higher direct and indirect costs based on semi-annual surveys of a tri-nation cohort of 715 systemic lupus erythematosus (SLE) patients over 4 years (269 US, 231 Canada, 215 United Kingdom [UK])^[[4]](#endnote-4)^. Based on this study, we derived direct and indirect costs for both remission and relapse states. In addition to costs related to pharmaceuticals, dialysis and major infections, the components of *direct* healthcare costs in our model included patient visits with specialists, non-specialists, non-physician health care professionals, laboratory studies, imaging studies, emergency room visits, outpatient surgery and hospitalizations^[[5]](#endnote-5)^.

The *indirect* costs in our model included time lost from labor and non-labor (i.e. household work) market activities^[[6]](#endnote-6)^. The indirect costs also included the time that a caregiver spent delivering health care to the patient, helping the patient in obtaining health care services or performing household tasks. In cost-effectiveness analysis, the costs of lost productivity are captured in the denominator of the cost-effectiveness ratio as QALYs and thus should not be double-counted by being placed in the numerator at the same time^[[7]](#endnote-7)^. From a societal perspective, the numerator should include the costs of health care services, time costs (costs of time spent in receiving the treatment) and costs borne by others i.e. caregivers. Clarke et al did not differentiate *time* costs from *productivity* costs in their analysis^4,6^. However, given the paucity of data in the literature, we incorporated Clarke’s data on indirect costs as best available information and conducted sensitivity analyses in which indirect costs have been excluded.

Clarke’s analysis also showed that patients in the US incurred 20% higher cumulative direct costs^5^ and 29% higher indirect costs^6^ than their Canadian counterparts. Thus, the *total* direct and indirect costs in our model likely underestimate the true health care expenditures from a US perspective. However, this cost disparity would not change the final results in our study given that the main outcome measure is based on incremental calculations between two strategies.

Utilities (QALY)

The utility scores for the remission and relapse health states were measured by a visual analog scale (VAS)^4,^ ^[[8]](#endnote-8)^. The VAS has been demonstrated to be a valid and reliable measure of health related quality of life in a SLE cohort^[[9]](#endnote-9)^. In our 3-year model, patients who require IV CYC after multiple relapses are modeled to have a lower utility score (0.5) compared to those who are treated with MMF for initial relapses (0.6). The lower score assigned to IV CYC was based on a study by Tse et al who showed that induction therapy with MMF was associated with better scores for all quality-of-life domains than CYC^[[10]](#endnote-10)^. Using the preference score of 0.69 for “typical severe sepsis” from the Tufts Cost-Effectiveness Analysis Registry, we derived a disutility of 0.31 for those patients who develop a major infection, representing its impact on quality of life (1 minus 0.69)^[[11]](#endnote-11),^ ^[[12]](#endnote-12)^. In the 3-year model, these individuals would incur this utility decrement, along with the excess costs, associated with a major infection.

Data Analysis

We used a two-dimensional simulation, combining second-order Monte Carlo probabilistic sensitivity analysis (PSA) in a first-order patient-level model^[[13]](#endnote-13)^. PSA represents the outer loop for parameter uncertainty, and microsimulation represents the inner loop for individual patient variability. In this two-loop simulation, all parameter distributions are first sampled. The sampled values then undergo 1,000 first-order trials which generate means for each sample value. This cycle of calculation is repeated until all parameter sampling is completed (1,000 PSA iterations). A discount rate of 3% per annum was applied to both costs and health outcomes. Half-cycle correction was applied to the model on the assumption that on average, transitions occur halfway through a cycle.

We used microsimulation to evaluate stochastic uncertainty, representing uncertainty in patient-level outcomes due to chance alone, while taking into account risk factors such as age in our model. For instance, we performed patient-level simulations of each individual’s starting age via a *uniform* distribution (20 to 40 years old) which would determine the background age-specific mortality rate. We used a uniform distribution to allow for equal probability of ages, in integers, bounded by 20 and 40 years, reflecting the mean ages of 33 ± 11 and 31 ± 11 in the MAINTAIN^[[14]](#endnote-14)^ and ALMS^[[15]](#endnote-15)^ trials, respectively.

We incorporated tracker variables in conjunction with microsimulation in our model to achieve “memory” of patients’ disease history. This modeling technique has several advantages. First, we used these trackers to keep count on the number of LN relapses, in order to determine the transition probability of whether to remain on the current rescue regimen vs. switching to CYC. For each strategy, it was assumed that patients who have been treated with MMF for 3 relapses (over 3 cycles or 18 months) were transitioned to CYC for up to 2 cycles. Thus the specific salvage regimen for each treatment arm was cycle-dependent. Second, we employed tracker variables to record and report clinical events to include death from lupus or other causes, major infection, ESRD and LN relapse. This data was used for external validation by comparing the model’s predicted results to actual event data. Third, we used microsimulation in our model to account for each individual’s age in order to determine the background age-specific mortality rate. Fourth, microsimulation generates means and standard deviations, as opposed to expected value calculations in Markov cohort analysis.

In addition to microsimulation, PSA simulations are conducted to assess the joint effect of multiple parameter uncertainties on our model. We used β distributions for both *probability* and *utility* parameters, γ distributions for *cost* parameters and lognormal distributions for *relative risk* parameters^[[16]](#endnote-16)^. Our PSA outputs included 1) cost-effectiveness acceptability curve (CEAC), which represents the probability of cost-effectiveness as a function of the threshold willingness-to-pay (WTP) by using net benefits to calculate the changing percentage of iterations for which a strategy is cost-effective relative to an alternative; 2) incremental cost-effectiveness (ICE) scatterplot, which includes individual points representing pairs of incremental cost and effectiveness values, based on one strategy relative to an alternative; 3) expected value of perfect information (EVPI). The EVPI value represents the expected cost of uncertainty; in other words, it is the expected value of collecting information in future research that would eliminate all parameter uncertainty^13^. We calculated both *total* and *population* EVPI, as noted in the next section.

Expected Value of Perfect Information (EVPI)

The *total* EVPI, estimated by the difference between the NMB value of the iteration’s optimal strategy and that of the baseline optimal strategy, was $0 per patient at WTP $50,000/QALY. At WTP $100,000/QALY, the total EVPI was $12.86 per patient. Thus, by eliminating all parameter uncertainty in the model, we can expect an incremental NMB of $12.86 per patient. The implication is that the current expected harm due to uncertainty is $12.86 per patient, with a health equivalent of 0.047 quality-adjusted life-days at WTP $100,000/QALY.

The *population* EVPI represents the upper bound on the expected gain on investment on further data collection (total EVPI/patient x population that is expected to benefit from future research). The incidence rate of LN among US adults with Medicaid coverage was 6.85 per 100,000 person-years^[[17]](#endnote-17)^. Generalizing to the overall US population and assuming a period of 10 years with 3% discount rate, we calculated a population EVPI of $2,058,206 at WTP $100,000/QALY. If this amount is higher than the expected costs of additional research then it may be cost effective to collect more data. This calculated population EVPI is likely overestimated given the higher LN burden in the low-income Medicaid cohort.

Sensitivity Analysis

We performed one-way sensitivity analyses, in which a single variable is tested over its plausible range with all other variables held constant, using microsimulation to account for stochastic (1st-order) uncertainty. In these analyses, individual sets of trials (10,000) are run through the model for each value of the variable of interest. We also performed a “tornado” analysis in which each variable is sequentially tested in a one-way sensitivity analysis, ranking them in order of overall influence on the expected value of the model^13^.

As scenario analyses represent an important component of sensitivity analysis ^[[18]](#endnote-18),^ ^[[19]](#endnote-19),^ ^[[20]](#endnote-20)^, we applied extreme values of the three most influential variables as derived from the “tornado” diagram, to simulate the least favorable conditions for the AZA group in the 3-year model.

Model Validation

We evaluated model validity according to established practice guidelines^[[21]](#endnote-21)^. As an initial assessment of *face validity*, the model that was constructed by the first author was put forth to the co-investigators of this study to evaluate the model components, assumptions, data sources and results. This peer review process was important in ensuring that the model was constructed in accordance to our current understanding of the disease process of LN and its treatment regimens.

*Internal validity* was evaluated by using extreme values of parameters in scenario analysis as well as one-way sensitivity analyses using broad range of input values to ensure that the model outputs (ICERs) were moving in the expected direction. These analyses were thus used to “debug” the model for any unintentional computational errors.

We tested *external validity* by comparing predicted results of the 3-year and lifetime models with actual event data. We evaluated *cross validation* by examining a different cost-utility model that addressed the same question but from a Thailand perspective^[[22]](#endnote-22)^.

Assessment of External Validity

1. 3-Year Model

In addition to comparing outputs from the 3-year model with observed data from the ALMS trial (Supplemental Table 7), we also compared predicted outcomes from our base-case model with a recent observational study of 61 patients treated for Class III-V LN in the United Kingdom, with a median follow-up of 68 months^[[23]](#endnote-23)^. This retrospective study showed that five of the 20 who used AZA (25.0%) and 10 of the 27 (37.0%) who used MMF had renal flares (p = 0.615). Our simulation predicted that 22% ± 11.5 of the patients on AZA and 12.2% ± 6.0 of those on MMF would relapse. Thus, the observed proportion of patients who relapsed on MMF was higher than the model’s predicted result. The authors of this retrospective study, however, acknowledged that the high number of relapse on MMF maintenance therapy may reflect a selection bias in which higher risk patients were preferentially treated with MMF.

1. Lifetime Model

We evaluated external validity of the lifetime model based on calculations of life years, without accounting for quality-of-life benefits. These calculations of life expectancy were non-discounted. We compared the 10-year follow-up data from the Euro-Lupus Nephritis Trial (ELNT)^[[24]](#endnote-24)^ with predicted outputs from our lifetime model. The ELNT is a randomized prospective trial comparing low dose vs. high dose intravenous CYC followed by maintenance therapy with AZA. At 10 years, of the 90 patients randomized in the clinical trial, 7 patients (7.8%) died and 6 patients (6.7%) reached ESRD. We used data from the MAINTAIN^14^ trial for predicted outputs, given the similar interventions and patient cohorts shared between MAINTAIN and ELNT. In a hypothetical cohort on AZA maintenance therapy, our simulations predicted that 5.2% ± 0.7 would die and 8.0% ± 1.0 would reach ESRD at 10 years. We then compared cumulative survival rates from our model with a retrospective study by Mok et al^[[25]](#endnote-25)^. Among the 694 Chinese SLE patients studied with a mean age of 32.9, the proportions of LN patients who had ever received AZA or MMF were 89% and 50%, respectively. Patients with LN in Mok’s study had a 10-year survival rate of 88.8%. Our lifetime model using Cochrane data predicted a 10-year survival rate of 90.4% ± 1.0 in those patients on AZA and 93.2% ± 1.0 in those on MMF. Overall, the predicted outcomes from the lifetime model approximated observed data from prior studies.

**Supplemental Table 1: Transition matrix table of MMF-based therapy using base-case (Cochrane) estimates in the three-year model, demonstrating transition probability from one health state to the next during each 6-month cycle^€^**

| State of next cycle | | | | | | | |
| --- | --- | --- | --- | --- | --- | --- | --- |
| State of current cycle |  | **Remission MMF 2gm/d** | **Relapse MMF 3gm/d** | **Relapse**  **IV CYC 0.75gm/m^2^** | **ESRD due to LN** | **Dead** | Total |
|  | **Remission MMF 2gm/d** | # | 0.01854^‡^ | n/a | 0.00118^‡^ | 0.00429^¥^ + pDeath_Other* | 1.0 |
|  | **Relapse MMF 3gm/d** | 0.59^‡^ | # | ¶ | 0.061^‡^ | 0.041^¥^ + pDeath_Other* | 1.0 |
|  | **Relapse**  **IV CYC 0.75gm/m^2^** | 0.522^‡^ | n/a | # | 0.0855^‡^ | 0.04^¥^ + pDeath_Other* | 1.0 |
|  | **ESRD due to LN** | n/a | n/a | n/a | # | 0.0513^£^ | 1.0 |
|  | **Dead** | n/a | n/a | n/a | n/a | 1.0 | 1.0 |

AZA: azathioprine; MMF: mycophenolate mofetil; CYC: cyclophosphamide; ESRD: end stage renal disease; LN: lupus nephritis; IV: intravenous; n/a: not applicable

^€^ Probabilities from the data sources were reported over various follow-up durations. *Probabilities* were converted to *rates* then to 6-month probabilities^[[26]](#endnote-26)^. First, the probabilities were converted to yearly rates (event per patient per year) using the equation:

$$r= -\frac{1}{t}ln \left( 1-P \right)$$

where r = rate; t = time in years; P = probability of an event occurring during time *t*.

These annual rates were then converted to 6-month probabilities using the equation:

$$P=1-e^{-rt}$$

where r = one-year rate; t = time in years; P = probability of an event occurring during time *t*.

#: complement of probability

^‡^ Cochrane 2012^2^

^¥^Probability of lupus-related death during health state^2^

*pDeath_Other: probability of death from other (non-LN) causes, which varies based on the age of the individual in the microsimulation trial and life table data^1^

^£^ Costenbader 2011^[[27]](#endnote-27)^

^¶^ Patients transition to IV CYC therapy for relapse only if > 3 total relapses (ie. if patients continue to relapse during the 3^rd^ year of treatment), otherwise patients remain on MMF 3gm/day for relapses

**Supplemental Table 2: Transition matrix table of AZA-based therapy using base-case (Cochrane) estimates in the three-year model, demonstrating transition probability from one health state to the next during each 6-month cycle^Δ^**

| State of next cycle | | | | | | | |
| --- | --- | --- | --- | --- | --- | --- | --- |
| State of current cycle |  | **Remission AZA 150mg/d** | **Relapse MMF 2gm/d** | **Relapse**  **IV CYC 0.75gm/m^2^** | **ESRD due to LN** | **Dead** | Total |
|  | **Remission AZA 150mg/d** | # | RR^€^ x 0.01854^†^ | n/a | RR^€^ x 0.00118^Ψ^ | [RR^€^ x 0.00429^¥^] + pDeath_Other* | 1.0 |
|  | **Relapse MMF 2gm/d** | 0.59^‡^ | # | ¶ | 0.061^‡^ | 0.041^¥^ + pDeath_Other* | 1.0 |
|  | **Relapse**  **IV CYC 0.75gm/m^2^** | n/a | 0.522^‡^ | # | 0.0855^‡^ | 0.04^§^ + pDeath_Other* | 1.0 |
|  | **ESRD due to LN** | n/a | n/a | n/a | # | 0.0513^£^ | 1.0 |
|  | **Dead** | n/a | n/a | n/a | n/a | 1.0 | 1.0 |

AZA: azathioprine; MMF: mycophenolate mofetil; CYC: cyclophosphamide; ESRD: end stage renal disease; LN: lupus nephritis; IV: intravenous; n/a: not applicable

^Δ^ Probabilities from the data sources were reported over various follow-up durations. *Probabilities* were converted to *rates* then to 6-month probabilities^26^. First, the probabilities were converted to yearly rates (event per patient per year) using the equation:

$$r= -\frac{1}{t}ln \left( 1-P \right)$$

where r = rate; t = time in years; P = probability of an event occurring during time *t*.

These annual rates were then converted to 6-month probabilities using the equation:

$$P=1-e^{-rt}$$

where r = one-year rate; t = time in years; P = probability of an event occurring during time *t*.

#: complement of probability

^‡^ Cochrane 2012^2^

*pDeath_Other: probability of death from other (non-LN) causes, which varies based on the age of the individual in the microsimulation trial and life table data^1^

^¶^ Patients transition to IV CYC therapy for relapse only if > 3 total relapses (ie. if patients continue to relapse during the 3^rd^ year of treatment), otherwise patients remain on MMF 2gm/day for relapses.

^¥^Probability of lupus-related death during health state on MMF^2^

^€^Relative risk of event on AZA compared to MMF^2^

^†^Probability of relapse during remission on MMF^2^

^Ψ^Probability of ESRD during remission on MMF^2^

^§^Probability of lupus-related death during relapse on CYC^2^

^£^ Costenbader 2011^27^

**Supplemental Table 3: Three-Year Model Inputs of Probability Parameters Based on ALMS Data**

| **Probability Parameters (Over 6-Month Period or One Cycle)^€^** | **Mean** | **Range (95% CI)** | **Probability Distribution**^#^ | **Sources** |
| --- | --- | --- | --- | --- |
|  |  |  |  |  |
| Remission AZA |  |  |  |  |
| Probability of lupus-related death during remission | 0.0015 | 0.0011-0.0019**^†^** | Beta (9.0, 5981.0) | ALMS 2011 (15) |
| Probability of major infection during remission | 0.0205 | 0.0152-0.0260**^†^** | Beta (16.4, 785.7) | ALMS 2011 (15) |
| Probability of ESRD during remission | 0.0046 | 0.0034-0.0057**^†^** | Beta (21.1, 4556.8) | ALMS 2011 (15) |
| Probability of relapse during remission | 0.0434 | 0.0214-0.0908 | Beta (18.0, 396.2) | ALMS 2011 (15) |
|  |  |  |  |  |
| Remission MMF |  |  |  |  |
| Probability of lupus-related death during remission | 0 | n/a | n/a | ALMS 2011 (15) |
| Probability of major infection during remission | 0.0167 | 0.0124-0.0211**^†^** | Beta (17.1, 1008.2) | ALMS 2011 (15) |
| Probability of ESRD during remission | 0 | n/a | n/a | ALMS 2011 (15) |
| Probability of relapse during remission | 0.0228 | 0.0115-0.0447 | Beta (20.3, 869.9) | ALMS 2011 (15) |
|  |  |  |  |  |
| Relapse MMF (2gm/d or 3gm/d) |  |  |  |  |
| Probability of lupus-related death during relapse | 0.0410 | 0.021-0.079 | Beta (64.4, 1507.3) | Cochrane 2012 (2) |
| Probability of major infection during relapse | 0.1210 | 0.081-0.183 | Beta (514.6, 3738.7) | Cochrane 2012 (2) |
| Probability of ESRD during relapse | 0.0610 | 0.023-0.158 | Beta (139.7, 2150.5) | Cochrane 2012 (2) |
| Probability of complete and partial remissions | 0.5900 | 0.418-0.738 | Beta (56.5, 39.3) | Cochrane 2012 (2) |
|  |  |  |  |  |
| Relapse CYC |  |  |  |  |
| Probability lupus-related death during relapse | 0.0400 | 0.0200-0.0780 | Beta (61.4, 1473.6) | Cochrane 2012 (2) |
| Probability of major infection during relapse | 0.1090 | 0.0730-0.1650 | Beta (105.8, 864.4) | Cochrane 2012 (2) |
| Probability of ESRD during relapse | 0.0855 | 0.0320-0.2220 | Beta (66.8, 714.1) | Cochrane 2012 (2) |
| Probability of complete and partial remissions | 0.5220 | 0.3920-0.6520 | Beta (51.6, 47.2) | Cochrane 2012 (2) |
|  |  |  |  |  |
| ESRD due to lupus nephritis |  |  |  |  |
| Probability of death due to lupus nephritis ESRD | 0.0513 | 0.0481-0.0548 | Beta (99.8, 1845.9) | Costenbader 2011 (27) |

AZA: azathioprine; MMF: mycophenolate mofetil; CYC: cyclophosphamide; ESRD: end stage renal disease; CI: confidence interval; n/a: not applicable

^€^ Probabilities from the data sources were reported over various follow-up durations. *Probabilities* were converted to *rates* then to 6-month probabilities^26^. First, the probabilities were converted to yearly rates (event per patient per year) using the equation:

$$r= -\frac{1}{t}ln \left( 1-P \right)$$

where r = rate; t = time in years; P = probability of an event occurring during time *t*.

These annual rates were then converted to 6-month probabilities using the equation:

$$P=1-e^{-rt}$$

where r = one-year rate; t = time in years; P = probability of an event occurring during time *t*.

**^†^** The ALMS study did not report 95% confidence intervals for these probability parameters; thus, we assumed ± 25% range

^#^ Beta distributions are characterized by (α, β)**Supplemental Table 4: Three-Year Model Inputs of Probability Parameters Based on MAINTAIN Data**

| **Probability Parameters (Over 6-Month Period or One Cycle)^€^** | **Mean** | **Range (95% CI)** | **Probability Distribution**^#^ | **Sources** |
| --- | --- | --- | --- | --- |
|  |  |  |  |  |
| Remission AZA |  |  |  |  |
| Probability of lupus-related death during remission | 0 | n/a | n/a | MAINTAIN 2010 (14) |
| Probability of major infection during remission | 0.0152 | 0.0112-0.0193**^†^** | Beta (25.3, 1636.9) | MAINTAIN 2010 (14) |
| Probability of ESRD during remission | 0.0024 | 0.0018-0.0030**^†^** | Beta (23.0, 9553.0) | MAINTAIN 2010 (14) |
| Probability of relapse during remission | 0.0353 | 0.0145-0.1001 | Beta (48.0, 1313.1) | MAINTAIN 2010 (14) |
|  |  |  |  |  |
| Remission MMF |  |  |  |  |
| Probability of lupus-related death during remission | 0.0048 | 0.0036-0.0060**^†^** | Beta (22.9, 4753.0) | MAINTAIN 2010 (14) |
| Probability of major infection during remission | 0.0176 | 0.0130-0.0223**^†^** | Beta (19.0, 1060.6) | MAINTAIN 2010 (14) |
| Probability of ESRD during remission | 0.0024 | 0.0017-0.0028**^†^** | Beta (23.0, 9553.0) | MAINTAIN 2010 (14) |
| Probability of relapse during remission | 0.0260 | 0.0109-0.0685 | Beta (26.3, 985.6) | MAINTAIN 2010 (14) |
|  |  |  |  |  |
| Relapse MMF (2gm/d or 3gm/d) |  |  |  |  |
| Probability of lupus-related death during relapse | 0.0410 | 0.0210-0.0790 | Beta (64.4, 1507.3) | Cochrane 2012 (2) |
| Probability of major infection during relapse | 0.1210 | 0.0810-0.1830 | Beta (514.6, 3738.7) | Cochrane 2012 (2) |
| Probability of ESRD during relapse | 0.0610 | 0.0230-0.1580 | Beta (139.7, 2150.5) | Cochrane 2012 (2) |
| Probability of complete and partial remissions | 0.5900 | 0.4180-0.7380 | Beta (56.5, 39.3) | Cochrane 2012 (2) |
|  |  |  |  |  |
| Relapse CYC |  |  |  |  |
| Probability lupus-related death during relapse | 0.0400 | 0.0200-0.0780 | Beta (61.4, 1473.6) | Cochrane 2012 (2) |
| Probability of major infection during relapse | 0.1090 | 0.0730-0.1650 | Beta (105.8, 864.4) | Cochrane 2012 (2) |
| Probability of ESRD during relapse | 0.0855 | 0.0320-0.2220 | Beta (66.8, 714.1) | Cochrane 2012 (2) |
| Probability of complete and partial remissions | 0.5220 | 0.3920-0.6520 | Beta (51.6, 47.2) | Cochrane 2012 (2) |
|  |  |  |  |  |
| ESRD due to lupus nephritis |  |  |  |  |
| Probability of death due to lupus nephritis ESRD | 0.0513 | 0.0481-0.0548 | Beta (99.8, 1845.9) | Costenbader 2011 (27) |

AZA: azathioprine; MMF: mycophenolate mofetil; CYC: cyclophosphamide; ESRD: end stage renal disease; CI: confidence interval; n/a: not applicable

^€^ Probabilities from the data sources were reported over various follow-up durations. *Probabilities* were converted to *rates* then to 6-month probabilities^26^. First, the probabilities were converted to yearly rates (event per patient per year) using the equation:

$$r= -\frac{1}{t}ln \left( 1-P \right)$$

where r = rate; t = time in years; P = probability of an event occurring during time *t*.

These annual rates were then converted to 6-month probabilities using the equation:

$$P=1-e^{-rt}$$

where r = one-year rate; t = time in years; P = probability of an event occurring during time *t*.

**^†^** The MAINTAIN study did not report 95% confidence intervals for these probability parameters; thus, we assumed ± 25% range

^#^ Beta distributions are characterized by (α, β)

**Supplemental Table 5: Relative risk parameters of AZA vs. MMF therapy, based on Cochrane data**

| **Relative Risk Parameters (Treatment Effect)** | **Mean Values** | **Range (95% CI)** | **Probability Distribution^†^** | **Sources** |
| --- | --- | --- | --- | --- |
|  |  |  |  |  |
| RR of death on AZA vs. MMF | 0.58 | 0.10-3.49 | Lognormal  (-0.545, 0.906) | Cochrane 2012 (2) |
| RR of major infection on AZA vs. MMF | 0.87 | 0.31-2.43 | Lognormal  (-0.139, 0.525) | Cochrane 2012 (2) |
| RR of ESRD on AZA vs. MMF | 1.86 | 0.37-9.31 | Lognormal  (0.620, 0.823) | Cochrane 2012 (2) |
| RR of relapse on AZA vs. MMF | 1.83 | 1.24-2.71 | Lognormal  (0.604, 0.199) | Cochrane 2012 (2) |

RR: relative risk; AZA: azathioprine; MMF: mycophenolate mofetil; ESRD: end stage renal disease; CI: confidence interval

^†^Lognormal distributions are characterized by (μ, σ); μ = log mean; σ = log standard error

**Supplemental Table 6: Costs of individual components of intravenous cyclophosphamide infusion‡**

| **Key Components of Cyclophosphamide Infusion** | **Mean cost per infusion per month ($)** | **Total cost per 6 months ($)** | **References** |
| --- | --- | --- | --- |
|  |  |  |  |
| Cyclophosphamide (IV) 0.75gm/m^2^ | 537.63^€^ | 3225.78 | Red Book 2013 (^[[28]](#endnote-28)^) |
| Mesna (IV) | 30.60^&^ | 183.60 | Red Book 2013 (28 ) |
| 1 liter 0.9% normal saline | 3.18 | 19.08 | Red Book 2013 (28 ) |
| Ondansetron (oral) | 162.32^†^ | 973.92 | Red Book 2013 (28) |
| IV infusion chemotherapeutic agent 1 hour (HCPCS 96413) | 230.50 | 1383.00 | CMS 2013 (^[[29]](#endnote-29)^) |
| IV hydration infusion (HCPCS 96360) | 74.69 | 448.14 | CMS 2013 (29) |
|  |  |  |  |
| Total | 1038.92 | 6233.52 |  |

IV: intravenous; HCPCS: Healthcare Common Procedure Coding System

^‡^ Excluded:

1. Intravenous methylprednisolone and oral prednisone
2. Proton-pump inhibitor: prophylaxis for steroid-induced gastritis and peptic ulcer disease
3. Trimethoprim/sulfamethoxazole: prophylaxis for Pneumocystis jirovecii pneumonia
4. Calcium/vitamin D: for steroid-induced osteoporosis
5. Leuprolide to decrease risk of ovarian failure

^€^ Mean cost of 1gm IV Cyclophosphamide = $413.56. Assuming an average adult with body surface area of 1.73m2, the total dose per infusion is ~1.3gm (0.75gm/m^2^ x 1.73m2). Total cost per infusion per month is $537.63 ($413.56/gm x 1.3gm)

^&^ Mean cost of IV Mesna 100mg/ml = $3.92; total Mesna dose is 60% of CYC dose (0.6 x 1300mg) = 780mg; $3.92 x 7.8 = $30.60

^†^Mean cost of 8mg tablet ondansetron = $40.58; 4 doses over 2 days = $162.32

**Supplemental Table 7: External validation, comparison of observed ALMS trial data with predicted outputs from the three-year model**

| **Trial Outcomes** | **Observed percentage of cohort (%)^15^** | **Predicted percentage of cohort (% ± standard deviation)** |
| --- | --- | --- |
|  |  |  |
| MMF |  |  |
| Renal flare | 12.9 | 15.57 ± 3.70 |
| Major infections | 9.6 | 9.47 ± 2.18 |
| ESRD | 0 | 0.70 ± .31 |
| Mortality | 0 | 0.51 ± .25 |
|  |  |  |
| AZA |  |  |
| Renal flare | 23.4 | 28.19 ± 6.75 |
| Major infections | 11.7 | 12.1 ± 2.64 |
| ESRD | 2.7 | 3.39 ± .80 |
| Mortality | 0.9 | 1.61 ± .49 |
|  |  |  |

**Supplemental Table 8: Transition matrix table of MMF-based therapy using base-case (Cochrane) estimates in the lifetime model, demonstrating transition probability from one health state to the next during each 1-year cycle^€^**

| State of next cycle | | | | | | |
| --- | --- | --- | --- | --- | --- | --- |
| State of current cycle |  | **Remission MMF** | **Relapse MMF** | **ESRD due to LN** | **Dead** | Total |
|  | **Remission MMF** | # | 0.0367^‡^ | 0.0025^‡^ | pDeath_Lupus^¥^ + pDeath_Other* | 1.0 |
|  | **Relapse MMF** | 0.8319^‡^ | # | 0.1183^‡^ | pDeath_Lupus^¥^ + pDeath_Other* | 1.0 |
|  | **ESRD due to LN** | n/a | n/a | # | pDeath_ESRD_LN^£^ | 1.0 |
|  | **Dead** | n/a | n/a | n/a | 1.0 | 1.0 |

AZA: azathioprine; MMF: mycophenolate mofetil; ESRD: end stage renal disease; LN: lupus nephritis; n/a: not applicable

^€^ Probabilities from the data sources were reported over various follow-up durations. *Probabilities* were converted to *rates* then to 6-month probabilities^26^. First, the probabilities were converted to yearly rates (event per patient per year) using the equation:

$$r= -\frac{1}{t}ln \left( 1-P \right)$$

where r = rate; t = time in years; P = probability of an event occurring during time *t*.

These annual rates were then converted to 6-month probabilities using the equation:

$$P=1-e^{-rt}$$

where r = one-year rate; t = time in years; P = probability of an event occurring during time *t*.

# complement of probability

^‡^ Cochrane 2012^2^

^¥^ pDeath_Lupus: age-specific probability of lupus related death during health state (see Supplemental Tables 10 & 11 and footnotes for derivation)^1,^ ^2,^ ^[[30]](#endnote-30)^

*pDeath_Other: probability of death from other (non-LN) causes, which varies based on the age of the individual in the microsimulation trial and life table data^1^

^£^ pDeath_ESRD_LN: age-specific probability of death due to lupus nephritis ESRD (see Supplemental Tables 10 & 11 and footnotes for derivation)^[[31]](#endnote-31),^ ^[[32]](#endnote-32)^

**Supplemental Table 9: Transition matrix table of AZA-based therapy using base-case (Cochrane) estimates in the lifetime model, demonstrating transition probability from one health state to the next during each 1-year cycle^€^**

| State of next cycle | | | | | | |
| --- | --- | --- | --- | --- | --- | --- |
| State of current cycle |  | **Remission AZA** | **Relapse AZA** | **ESRD due to LN** | **Dead** | Total |
|  | **Remission AZA** | # | 0.0716^‡^ | 0.0061^‡^ | pDeath_Lupus^¥^ + pDeath_Other* | 1.0 |
|  | **Relapse AZA** | 0.8319^‡^ | # | 0.1183^‡^ | pDeath_Lupus^¥^ + pDeath_Other* | 1.0 |
|  | **ESRD due to LN** | n/a | n/a | # | pDeath_ESRD_LN^£^ | 1.0 |
|  | **Dead** | n/a | n/a | n/a | 1.0 | 1.0 |

AZA: azathioprine; MMF: mycophenolate mofetil; CYC: cyclophosphamide; ESRD: end stage renal disease; LN: lupus nephritis; IV: intravenous; n/a: not applicable

^€^ Probabilities from the data sources were reported over various follow-up durations. *Probabilities* were converted to *rates* then to 6-month probabilities^26^. First, the probabilities were converted to yearly rates (event per patient per year) using the equation:

$$r= -\frac{1}{t}ln \left( 1-P \right)$$

where r = rate; t = time in years; P = probability of an event occurring during time *t*.

These annual rates were then converted to 6-month probabilities using the equation:

$$P=1-e^{-rt}$$

where r = one-year rate; t = time in years; P = probability of an event occurring during time *t*.

# complement of probability

^‡^ Cochrane 2012^2^

^¥^ pDeath_Lupus: age-specific probability of lupus related death during health state (see Supplemental Tables 10 & 11and footnotes for derivation)^1, 2, 30^

*pDeath_Other: probability of death from other (non-LN) causes, which varies based on the age of the individual in the microsimulation trial and life table data^1^

^£^ pDeath_ESRD_LN: age-specific probability of death due to lupus nephritis ESRD (see Supplemental Tables 10 & 11 and footnotes for derivation)^31,32^ **Supplemental Table 10: Lifetime Model Inputs of Probability Parameters based on ALMS Data**

| **Probability Parameters (1-year cycle)^€^** | **Mean Value** | **Range (95% CI)** | **Probability Distribution**^#^ | **Sources** |
| --- | --- | --- | --- | --- |
|  |  |  |  |  |
| Remission in AZA group |  |  |  |  |
| Probability of lupus-related death during remission | Age-dependent^3^ | n/a | n/a | Bernatsky 2006 (30); Arias 2007 (1); Cochrane 2012 (2) |
| Probability of ESRD during remission | 0.0091 | 0.0068-0.0114**^‡^** | Beta (326.8, 35,662.4) | ALMS 2011 (15) |
| Probability of relapse during remission | 0.0850 | 0.0423-0.1734 | Beta (26,443.4, 284,655.6) | ALMS 2011 (15) |
|  |  |  |  |  |
| Remission in MMF group |  |  |  |  |
| Probability of lupus-related death during remission | Age-dependent^2^ | n/a | n/a | Bernatsky 2006 (30); Arias 2007 (1); Cochrane 2012 (2) |
| Probability of ESRD during remission | 0 | n/a | n/a | ALMS 2011 (15) |
| Probability of relapse during remission | 0.0450 | 0.0229-0.0874 | Beta (23.8, 505.7) | ALMS 2011 (15) |
|  |  |  |  |  |
| Relapse in MMF group |  |  |  |  |
| Probability of lupus-related death during relapse | Age-dependent^1^ | n/a | n/a | Bernatsky 2006 (30); Arias 2007 (1) |
| Probability of ESRD during relapse | 0.1183 | 0.0455-0.2910 | Beta  (491.1, 3670.9) | Cochrane 2012 (2) |
| Probability of complete and partial remissions | 0.8319 | 0.6613-0.9313 | Beta  (45.7, 9.2) | Cochrane 2012 (2) |
|  |  |  |  |  |
| Relapse in AZA group |  |  |  |  |
| Probability lupus-related death during relapse | Age-dependent^1^ | n/a | n/a | Bernatsky 2006 (30); Arias 2007 (1) |
| Probability of ESRD during relapse | 0.1183^†^ | 0.0455-0.2910 | Beta  (491.1, 3670.9) | Cochrane 2012 (2) |
| Probability of complete and partial remissions | 0.8319^†^ | 0.6613-0.9313 | Beta  (45.7, 9.2) | Cochrane 2012 (2) |
|  |  |  |  |  |
| ESRD due to lupus nephritis |  |  |  |  |
| Probability of death due to lupus nephritis ESRD | Age-dependent^§^ | n/a | n/a | USRDS 2012 (31); Sule 2011 (32) |

AZA: azathioprine; MMF: mycophenolate mofetil; ESRD: end stage renal disease; CI: confidence interval; ALMS: Aspreva Lupus Management Study; n/a: not applicable

^€^ Probabilities from the data sources were reported over various follow-up durations. *Probabilities* were converted to *rates* then to 6-month probabilities^26^. First, the probabilities were converted to yearly rates (event per patient per year) using the equation:

$$r= -\frac{1}{t}ln \left( 1-P \right)$$

where r = rate; t = time in years; P = probability of an event occurring during time *t*.

These annual rates were then converted to 6-month probabilities using the equation:

$$P=1-e^{-rt}$$

where r = one-year rate; t = time in years; P = probability of an event occurring during time *t*.

**^‡^** The ALMS study did not report 95% confidence intervals for this probability parameter; thus, we assumed ± 25% range

^#^ Beta distributions are characterized by (α, β)

†probability based on MMF for relapse in either AZA or MMF-based regimen

^§^ The age-specific annual mortality rate for the general dialysis population in 2011^31^ is multiplied by hazard ratio (HR) 1.7. In a USRDS study, Sule et al found that adult patients with ESRD secondary to SLE were at increased risk of death compared with other adult patients (HR 1.7; 95% CI 1.2-2.7)^32^. Conversion between rates and probabilities as noted above.

^1^ In the relapse state for both MMF and AZA strategies, the rate of lupus-related death is derived from age-specific annual mortality rate in the general population^1^ multiplied by a standardized mortality ratio (SMR) 7.9. In a cohort of 9,547 SLE patients, Bernatsky et al estimated an SMR 7.9 in those with nephritis^30^. Conversion between rates and probabilities as noted above.

^2^ Values in (1) divided by 9.3, given that the relative risk of lupus-related death during relapse vs. remission on MMF treatment is 9.3^2^.

^3^ Values in (2) x 0.58, given that the relative risk of lupus-related death during remission on AZA vs. MMF is 0.58^2^.

**Supplemental Table 11: Lifetime Model Inputs of Probability Parameters Based on MAINTAIN Data**

| **Probability Parameters (1-year cycle)^€^** | **Mean Value** | **Range (95% CI)** | **Probability**  **Distribution**^#^ | **Sources** |
| --- | --- | --- | --- | --- |
|  |  |  |  |  |
| Remission in AZA group |  |  |  |  |
| Probability of lupus-related death during remission | Age-dependent^3^ | n/a | n/a | Bernatsky 2006 (30); Arias 2007 (1); Cochrane 2012 (2) |
| Probability of ESRD during remission | 0.0048 | 0.0036-0.0061**^‡^** | Beta (91.7, 19,015.1) | MAINTAIN 2010 (14) |
| Probability of relapse during remission | 0.0694 | 0.0287-0.1902 | Beta (17,928.3, 240,405.2) | MAINTAIN 2010 (14) |
|  |  |  |  |  |
| Remission in MMF group |  |  |  |  |
| Probability of lupus-related death during remission | Age-dependent^2^ | n/a | n/a | Bernatsky 2006 (30); Arias 2007 (1); Cochrane 2012 (2) |
| Probability of ESRD during remission | 0.0048 | 0.0036-0.0059**^‡^** | Beta (91.7, 19,015.1) | MAINTAIN 2010 (14) |
| Probability of relapse during remission | 0.0513 | 0.0216-0.1323 | Beta (30.8, 569.1) | MAINTAIN 2010 (14) |
|  |  |  |  |  |
| Relapse in MMF group |  |  |  |  |
| Probability of lupus-related death during relapse | Age-dependent^1^ | n/a | n/a | Bernatsky 2006 (30); Arias 2007 (1) |
| Probability of ESRD during relapse | 0.1183 | 0.0455-0.2910 | Beta  (491.1, 3670.9) | Cochrane 2012 (2) |
| Probability of complete and partial remissions | 0.8319 | 0.6613-0.9313 | Beta  (45.7, 9.2) | Cochrane 2012 (2) |
|  |  |  |  |  |
| Relapse in AZA group |  |  |  |  |
| Probability lupus-related death during relapse | Age-dependent^1^ | n/a | n/a | Bernatsky 2006 (30); Arias 2007 (1) |
| Probability of ESRD during relapse | 0.1183^†^ | 0.0455-0.2910 | Beta  (491.1, 3670.9) | Cochrane 2012 (2) |
| Probability of complete and partial remissions | 0.8319^†^ | 0.6613-0.9313 | Beta  (45.7, 9.2) | Cochrane 2012 (2) |
|  |  |  |  |  |
| ESRD due to lupus nephritis |  |  |  |  |
| Probability of death due to lupus nephritis ESRD | Age-dependent^§^ | n/a | n/a | USRDS 2012 (31); Sule 2011 (32) |

AZA: azathioprine; MMF: mycophenolate mofetil; ESRD: end stage renal disease; CI: confidence interval; n/a: not applicable

^€^ Probabilities from the data sources were reported over various follow-up durations. *Probabilities* were converted to *rates* then to 6-month probabilities^26^. First, the probabilities were converted to yearly rates (event per patient per year) using the equation:

$$r= -\frac{1}{t}ln \left( 1-P \right)$$

where r = rate; t = time in years; P = probability of an event occurring during time *t*.

These annual rates were then converted to 6-month probabilities using the equation:

$$P=1-e^{-rt}$$

where r = one-year rate; t = time in years; P = probability of an event occurring during time *t*.

**^‡^** The MAINTAIN study did not report 95% confidence intervals for these probability parameters; thus, we assumed ± 25% range

^#^ Beta distributions are characterized by (α, β)

†probability based on MMF for relapse in either AZA or MMF-based regimen

^§^ The age-specific annual mortality rate for the general dialysis population in 2011^31^ is multiplied by hazard ratio (HR) 1.7. In a USRDS study, Sule et al found that adult patients with ESRD secondary to SLE were at increased risk of death compared with other adult patients (HR 1.7; 95% CI 1.2-2.7)^32^. Conversion between rates and probabilities as noted above.

^1^ In the relapse state for both MMF and AZA strategies, the rate of lupus-related death is derived from age-specific annual mortality rate in the general population^1^ multiplied by a standardized mortality ratio (SMR) 7.9. In a cohort of 9,547 SLE patients, Bernatsky et al estimated an SMR 7.9 in those with nephritis^30^. Conversion between rates and probabilities as noted above.

^2^ Values in (1) divided by 9.3, given that the relative risk of lupus-related death during relapse vs. remission on MMF treatment is 9.3^2^.

^3^ Values in (2) x 0.58, given that the relative risk of lupus-related death during remission on AZA vs. MMF is 0.58^2^.


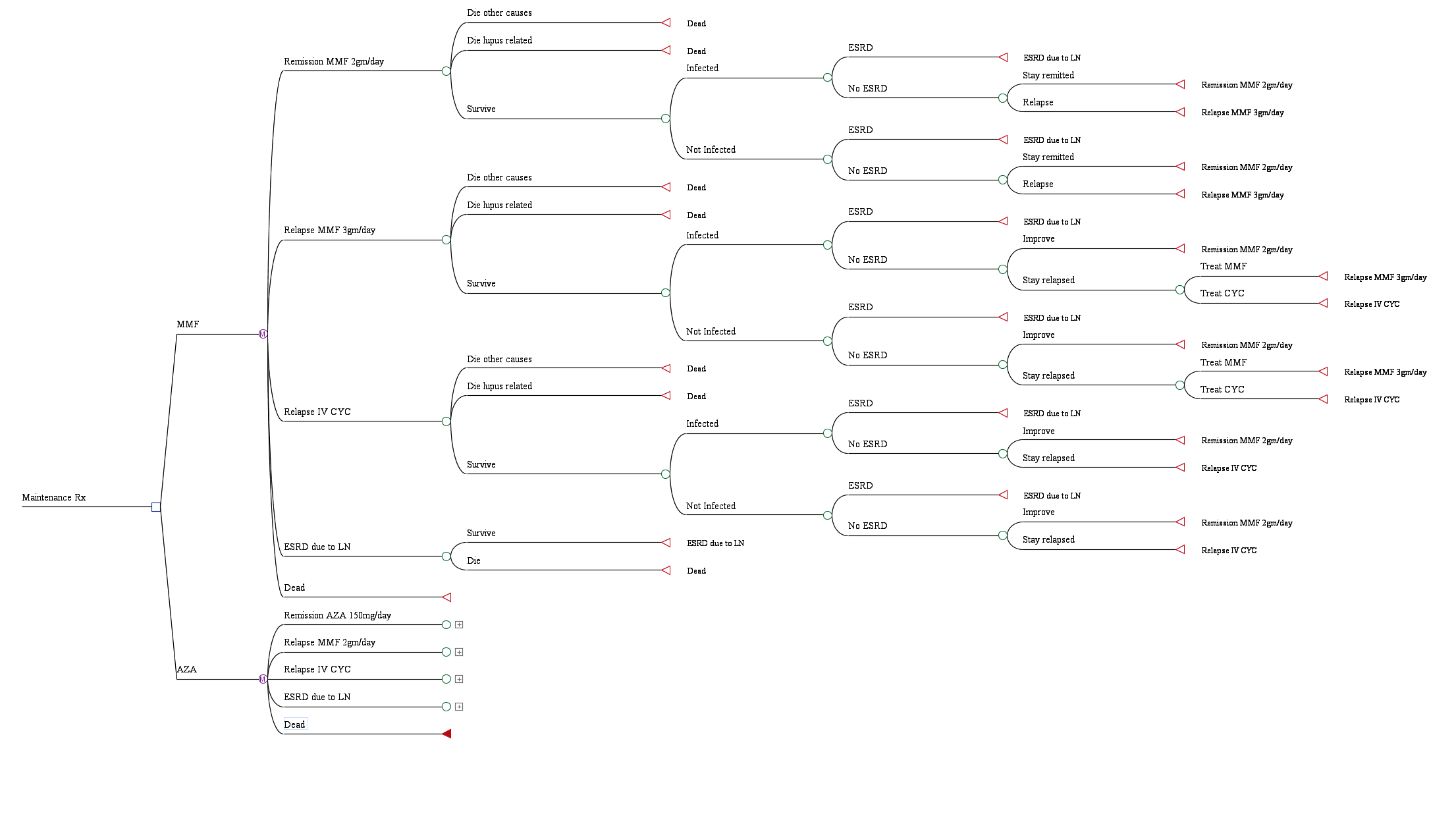


**Supplemental Figure 1:** Markov cycle tree of the three-year model, accounting for relapses and rescue therapy, major infections, progression to ESRD and deaths (both LN and non-LN related).  The subtrees emanating from the chance nodes in the AZA group are collapsed and represented by the + sign. LN: lupus nephritis; AZA: azathioprine; MMF: mycophenolate mofetil; IV CYC: intravenous cyclophosphamide; ESRD: end stage renal disease.

**Supplemental Figure 2:** Markov cycle tree of the lifetime model. LN: lupus nephritis; AZA: azathioprine; MMF: mycophenolate mofetil; ESRD: end stage renal disease.


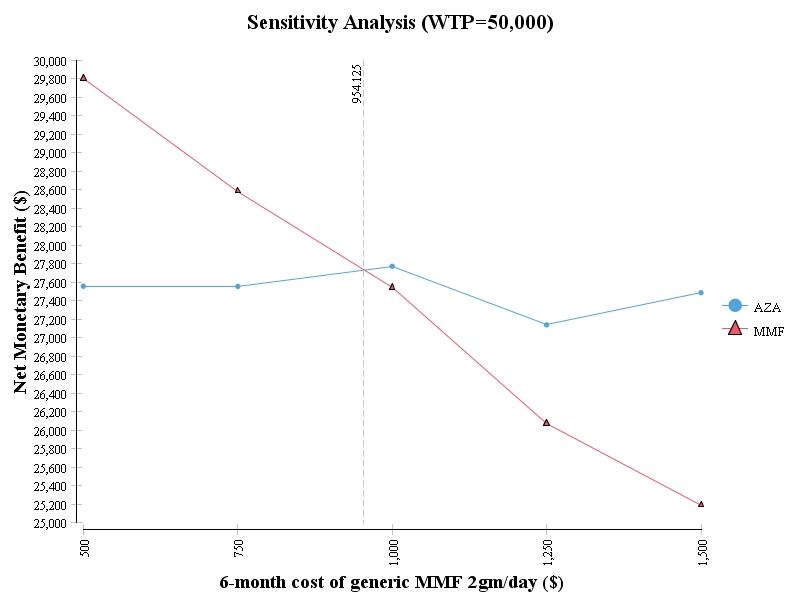


**Supplemental Figure 3:** One-way sensitivity analysis @ WTP $50,000/QALY based on the 3-year base-case model. It evaluates the effects of varying the costs of generic MMF per 6-month cycle on net monetary benefit over 10,000 microsimulation trials. WTP = willingness-to-pay; AZA: azathioprine; MMF: mycophenolate mofetil.


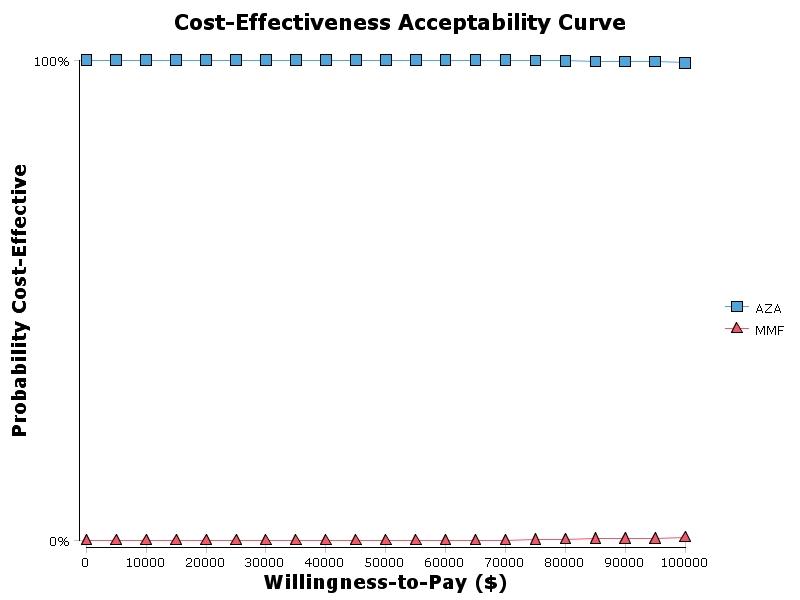


**Supplemental Figure 4**: Cost-effectiveness acceptability curve of the 3-year base-case model, generated from probabilistic sensitivity analyses via second-order Monte Carlo simulation of 1,000 iterations. It represents the probability of cost-effectiveness of the two strategies, as a function of the threshold willingness-to-pay (WTP). Near 100% of the iterations demonstrate AZA as cost-effective compared to an MMF-based strategy @ WTP $50,000 and $100,000/QALY over a 3-year time frame. AZA: azathioprine; MMF: mycophenolate mofetil

**
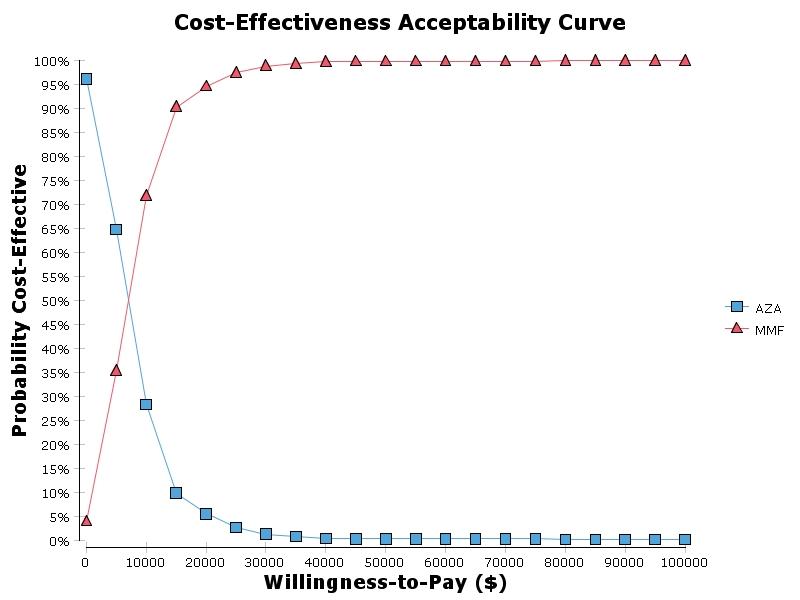
**

**Supplemental Figure 5**: Cost-effectiveness acceptability curve of the lifetime base-case (40-year) model, generated from probabilistic sensitivity analyses via second-order Monte Carlo simulation of 1,000 iterations. It represents the probability of cost-effectiveness of the two strategies, as a function of the threshold WTP. Near 100% of the iterations demonstrate MMF as cost-effective compared to an AZA-based strategy @ WTP $50,000 and $100,000/QALY (in contrast to the 3-year model as shown in Supplemental Figure 4). AZA: azathioprine; MMF: mycophenolate mofetil

1. Arias E. United States life tables, 2007. *Natl Vital Stat Rep* 2011; 59: 1-60. [↑](#endnote-ref-1)
2. Henderson L, Masson P, Craig JC, et al. Treatment for lupus nephritis. *Cochrane Database Syst Rev* 2012; 12: CD002922. [↑](#endnote-ref-2)
3. Contreras G, Pardo V, Leclercq B, et al. Sequential therapies for proliferative lupus nephritis. *N Engl J Med* 2004; 350: 971-80. [↑](#endnote-ref-3)
4. Clarke AE, Panopalis P, Petri M, et al. SLE patients with renal damage incur higher health care costs. *Rheumatology* (Oxford) 2008; 47: 329-33. [↑](#endnote-ref-4)
5. Clarke AE, Petri M, Manzi S, et al. The systemic lupus erythematosus Tri-nation Study: absence of a link between health resource use and health outcome. *Rheumatology* (Oxford) 2004; 43: 1016-24. [↑](#endnote-ref-5)
6. Panopalis P, Petri M, Manzi S, et al. The systemic lupus erythematosus Tri-Nation study: cumulative indirect costs. *Arthritis Rheum* 2007; 57: 64-70. [↑](#endnote-ref-6)
7. Weinstein MC, Siegel JE, Gold MR, Kamlet MS, Russell LB. Recommendations of the Panel on Cost-effectiveness in Health and Medicine*. JAMA* 1996; 276: 1253-8. [↑](#endnote-ref-7)
8. Grootscholten C, Snoek FJ, Bijl M, van Houwelingen HC, Derksen RH, Berden JH; Dutch Working Party of SLE. Health-related quality of life and treatment burden in patients with proliferative lupus nephritis treated with cyclophosphamide or azathioprine/ methylprednisolone in a randomized controlled trial. *J Rheumatol* 2007; 34: 1699-707. [↑](#endnote-ref-8)
9. Moore AD, Clarke AE, Danoff DS, et al. Can health utility measures be used in lupus research? A comparative validation and reliability study of 4 utility indices. *J Rheumatol* 1999; 26: 1285-90. [↑](#endnote-ref-9)
10. Tse KC, Tang CS, Lio WI, Lam MF, Chan TM. Quality of life comparison between corticosteroid- and-mycofenolate mofetil and corticosteroid- and-oral cyclophosphamide in the treatment of severe lupus nephritis. *Lupus* 2006; 15: 371-9. [↑](#endnote-ref-10)
11. Tufts’ Cost-Effectiveness Analysis Registry. https://research.tufts nemc.org/cear4/ Home.aspx (accessed 13 December 2013). [↑](#endnote-ref-11)
12. Wilson EC, Jayne DR, Dellow E, Fordham RJ. The cost-effectiveness of mycophenolate mofetil as firstline therapy in active lupus nephritis. *Rheumatology* (Oxford) 2007; 46: 1096-101. [↑](#endnote-ref-12)
13. Groot Koerkamp B, Weinstein MC, Stijnen T, Heijenbrok-Kal MH, Hunink MG.Uncertainty and patient heterogeneity in medical decision models. *Med Decis Making* 2010; 30: 194-205. [↑](#endnote-ref-13)
14. Houssiau FA, D'Cruz D, Sangle S, et al; MAINTAIN Nephritis Trial Group. Azathioprine versus mycophenolate mofetil for long-term immunosuppression in lupus nephritis: results from the MAINTAIN Nephritis Trial. *Ann Rheum Dis* 2010; 69: 2083-9. [↑](#endnote-ref-14)
15. Dooley MA, Jayne D, Ginzler EM, et al; ALMS Group. Mycophenolate versus azathioprine as maintenance therapy for lupus nephritis*. N Engl J Med* 2011; 365: 1886-95. [↑](#endnote-ref-15)
16. Briggs AH, Goeree R, Blackhouse G, O'Brien BJ. Probabilistic analysis of cost-effectiveness models: choosing between treatment strategies for gastroesophageal reflux disease. *Med Decis Making* 2002; 22: 290-308. [↑](#endnote-ref-16)
17. Feldman CH, Hiraki LT, Liu J, et al. Epidemiology and sociodemographics of systemic lupus erythematosus and lupus nephritis among US adults with Medicaid coverage, 2000-2004. *Arthritis Rheum* 2013; 65: 753-63. [↑](#endnote-ref-17)
18. Briggs AH, Weinstein MC, Fenwick EA, Karnon J, Sculpher MJ, Paltiel AD; ISPOR-SMDM Modeling Good Research Practices Task Force. Model parameter estimation and uncertainty analysis: a report of the ISPOR-SMDM Modeling Good Research Practices Task Force Working Group-6. *Med Decis Making* 2012; 32: 722-32. [↑](#endnote-ref-18)
19. Halpern MT, Luce BR, Brown RE, Geneste B. Health and economic outcomes modeling practices: a suggested framework. *Value Health* 1998; 1: 131-47. [↑](#endnote-ref-19)
20. Nee R, Parker AL, Little DJ, et al. Cost-effectiveness of antiplatelet therapy to prolong primary patency of hemodialysis graft. *Clin Nephrol* 2014; 81: 38-51. [↑](#endnote-ref-20)
21. Eddy DM, Hollingworth W, Caro JJ, Tsevat J, McDonald KM, Wong JB; ISPOR-SMDM Modeling Good Research Practices Task Force. Model transparency and validation: a report of the ISPOR-SMDM Modeling Good Research Practices Task Force-7. *Med Decis Making* 2012; 32: 733-43. [↑](#endnote-ref-21)
22. Mohara A, Pérez Velasco R, Praditsitthikorn N, Avihingsanon Y, Teerawattananon Y. A cost-utility analysis of alternative drug regimens for newly diagnosed severe lupus nephritis patients in Thailand. *Rheumatology* (Oxford) 2014; 53: 138-44. [↑](#endnote-ref-22)
23. Hui M, Garner R, Rees F, et al. Lupus nephritis: a 15-year multi-centre experience in the UK. *Lupus* 2013; 22: 328-32. [↑](#endnote-ref-23)
24. Houssiau FA, Vasconcelos C, D'Cruz D, et al. The 10-year follow-up data of the Euro-Lupus

    Nephritis Trial comparing low-dose and high-dose intravenous cyclophosphamide. *Ann Rheum Dis* 2010; 69: 61-4. [↑](#endnote-ref-24)
25. Mok CC, Kwok RC, Yip PS. Effect of renal disease on the standardized mortality ratio and life expectancy of patients with systemic lupus erythematosus. *Arthritis Rheum* 2013; 65: 2154-60. [↑](#endnote-ref-25)
26. Sonnenberg FA, Beck JR. Markov models in medical decision making: a practical guide. *Med Decis Making* 1993; 13: 322-38. [↑](#endnote-ref-26)
27. Costenbader KH, Desai A, Alarcón GS, et al. Trends in the incidence, demographics, and outcomes of end-stage renal disease due to lupus nephritis in the US from 1995 to 2006. *Arthritis Rheum* 2011; 63: 1681-8. [↑](#endnote-ref-27)
28. Red Book Online. <http://www.redbook.com/redbook/online> (accessed 25 June 2013). [↑](#endnote-ref-28)
29. Centers for Medicare & Medicaid Services. [http://www.cms.gov/Medicare/ Medicare- Fee-for-Service Payment /HospitalOutpatientPPS/Addendum-A-and-Addendum-B-Updates.html](http://www.cms.gov/Medicare/%20Medicare-%20Fee-for-Service%20Payment%20/HospitalOutpatientPPS/Addendum-A-and-Addendum-B-Updates.html) (accessed 11 December 2013). [↑](#endnote-ref-29)
30. Bernatsky S, Boivin JF, Joseph L, et al. Mortality in systemic lupus erythematosus. *Arthritis Rheum* 2006; 54: 2550-7. [↑](#endnote-ref-30)
31. US Renal Data System, USRDS 2012 Annual Data Report: Atlas of Chronic Kidney Disease and End-Stage Renal Disease in the United States, National Institutes of Health, National Institute of Diabetes and Digestive and Kidney Diseases, Bethesda, MD, 2012. [↑](#endnote-ref-31)
32. Sule S, Fivush B, Neu A, Furth S. Increased risk of death in pediatric and adult patients with ESRD secondary to lupus. *Pediatr Nephrol* 2011; 26: 93-8. [↑](#endnote-ref-32)
